# Supplementary material for: Cohort profile: Health trajectories of Immigrant Children (CRIAS)–a prospective cohort study in the metropolitan area of Lisbon, Portugal
Source: BMJ Open. 2022 Oct 25;12(10):e061919. doi: 10.1136/bmjopen-2022-061919 (PMC9608527; doi:10.1136/bmjopen-2022-061919)
Supplement: Supplementary data [file bmjopen-2022-061919supp001.pdf]

**Supplementary table 1a.** Key health oucomes for children at age 4/5 years

| | Immigrant | Non-immigrant | Total | *p* value |
|---|---|---|---|---|
| | n (%) | n (%) | n (%) | |
| | 217 (51.7) | 203 (48.3) | 420 (100) | |
| **Perceived health by parents** n=415 | | | | 0.986* |
| Very good and good | 173 (80.1) | 160 (80.4) | 333 (80.2) | |
| Fair | 39 (18.1) | 35 (17.6) | 74 (17.8) | |
| Bad and very bad | 4 (1.9) | 4 (2.0) | 8 (1.9) | |
| **Illness episodes in previous 3 months (parent reported)** n=410 | | | | 0.340** |
| Median | 1 (0-5;1) | 1 (0-5;2) | 1(0-5;1) | |
| (min-max;IQR) | | | | |
| **Psychomotor development** n=281 | | | | |
| All items achieved | 88 (61.5) | 99 (71.7) | 187 (66.5) | 0.077* |
| To monitor | 55 (38.5) | 39 (28.3) | 94 (33.5) | |
| **Emotional and behavioural difficulties** n=420 | | | | |
| Median externalizing behaviours (min.-max.;IQR) | 7 (0-18; 5)[a] | 7.5 (1-20; 5) | 7 (0-20; 5) | 0.950** |
| Median internalizing behaviours (min.-max.;IQR) | 4 (0-13; 4) | 3 (0-15; 3) | 4 (0-15;4) | p<0.001** |
| **Vaccination up to date** n=416 | | | | p=0.852* |
| yes | 195 (91.1) | 183 (90.6) | 378 (90.9) | |
| no | 19 (8.9) | 19 (9.4) | 38 (9.1) | |

Significance level 5%. *Pearson- Chi square statistical test ** Mann-Whitney U statistical

[a] children born in a non-EU country median 9 (min.1-max.15;IQR5) for externalizing behaviours

**Supplementary table 1b.** Factors associated with the odds of developing emotional and behavioural difficulties at ages 4/5. Logistic regression model with immigrant status variable: 1st generation immigrant children.

| | **Adjusted Odds-Ratio** | **95% Confidence Interval** | ***p* value** |
|---|---|---|---|
| **Variables** | | | |
| **Gender of the child** | | | |
| Boy | 1.114 | 0.716–1.734 | 0.632 |
| Girl | reference | | |
| **Immigrant status** | | | |
| Child is 1st generation immigrant | 2.247 | 1.062–4.756 | 0.034 |
| Child is not 1st generation immigrant | reference | | |
| **Household monthly income** | | | |
| <500 € | 3.512 | 1.135–10.861 | 0.029 |
| >500–750 € | 1.968 | 0.705–492 | 0.196 |
| >750–1000 € | 1.947 | 0.682–5.562 | 0.213 |
| >1000–1500 € | 1.870 | 0.664–5.271 | 0.236 |
| >1500–2000 € | 1.531 | 0.469–5.001 | 0.480 |
| >2000 € | reference | | |
| **Parents Educational level** | | | |
| Lower education | 2.995 | 1.303–6.884 | 0.010 |
| 9 years schooling | 3.237 | 1.482–7.068 | 0.003 |
| between 9 and 12 years | 2.226 | 1.124–4.410 | 0.022 |
| University degree | reference | | |

Supplementary table 1c. Psychomotor development at age 5

| **Psychomotor development** n=107 | Immigrant | Non-Immigrant | Total | p value |
|---|---|---|---|---|
| Monitoring not required | 37 (66.1) | 40 (78.4) | 77 (72.0) | p=0.155* |
| Monitoring required | 19 (33.9) | 11 (21.6) | 30 (28.0) | |

Significance level 5%. *Pearson- Chi square statistical test
