# Supplementary material for: Cohort profile: Health trajectories of Immigrant Children (CRIAS)–a prospective cohort study in the metropolitan area of Lisbon, Portugal
Source: BMJ Open. 2022 Oct 25;12(10):e061919. doi: 10.1136/bmjopen-2022-061919 (PMC9608527; doi:10.1136/bmjopen-2022-061919)
Supplement: Supplementary data [file bmjopen-2022-061919supp002.pdf]

Supplementary table 2. Fruit and vegetable intake and Body Mass Index (BMI) of children in the CRIAS cohort at ages 4/5

**Variables**	**Immigrant**	**Native**	**Total**	**p Value**
	n (%)	n (%)	n (%)	
	217 (51.7)	203 (48.3)	420 (100)	
**Dietary intake [a]**				
**Servings of fruit per day n=412**				<0.001*
Less than 2 servings/day	76 (36.0)	41 (20.4)	117 (27.9)	
2 or more servings/day	135 (64.0)	60 (79.6)	295 (70.2)	
**Servings of vegetables per day n=413**				<0.001*
Less than 3 servings/day	203 (95.8)	195 (97.0)	398 (94.8)	
3 or more servings/day	9 (4.2)	6 (3.0)	15 (3.6)	
**BMI n=314 [b]**				
**Overweight**				0.216*
Yes	35 (22.2)	44 (28.2)	79 (25.2)	
No	112 (71.8)	123 (77.8)	235 (74.8)	
**Obesity**				0.824*
Yes	9 (5.1)	8 (5.7)	17 (5.4)	
No	149 (94.9)	148 (94.3)	297 (94.6)	
**Underweight**				0.044*
Yes	18 (11.4)	8 (5.1)	26 (8.3)	
No	148 (88.6)	140	288 (91.7)	

Significance level 5%. *Pearson- Chi square statistical test

[a] intake according to recommend servings/day by the Portuguese Health Directorate
[b] underweight, overweight and obesity were classified using the World Health Organization Child Growth Standards charts
