# Supplementary material for: Cohort profile: Health trajectories of Immigrant Children (CRIAS)–a prospective cohort study in the metropolitan area of Lisbon, Portugal
Source: BMJ Open. 2022 Oct 25;12(10):e061919. doi: 10.1136/bmjopen-2022-061919 (PMC9608527; doi:10.1136/bmjopen-2022-061919)
Supplement: Supplementary data [file bmjopen-2022-061919supp003.pdf]

Supplementary table 3. Socioeconomic status and COVID-19 economic impact on families in the CRIAS-cohort study

	Non-Immigrants %	Immigrants %	Crude Odds-ratio (95% CI)	Adjusted Odds-ratio (95% CI)
**Education**				
Professional and higher education	30.9	17.1	1 [reference]	1 [reference]
Secondary education	35.5	38.8	2.0 (1.1-3.7)	1.18 (0.59-2.36)
Less than secondary education	33.6	44.0	2.4 (1.3-4.4)	0.89 (0.42-1.89)
**Employment**				
Employed	82.2	64.4	1 [reference]	1 [reference]
Unemployed and others	17.8	35.6	2.55 (1.48-4.41)	1.92 (1.03-3.55)
**Occupation**				
High-skilled occupations	81.6	57.8	1 [reference]	1 [reference]
Low-skilled occupations	18.4	42.2	3.24 (1.90-5.52)	2.49 (1.35-4.60)
**Family income before the pandemic**				
≥ 750 Euros	73.6	47.7	1 [reference]	1 [reference]
< 750 Euros	26.4	52.3	3.03 (1.84-5.09)	2.41 (1.36-4.28)
**Unemployed because of COVID-19**				
No	90.1	73.3	1 [reference]	1 [reference]
Yes	9.9	26.7	3.32 (1.72-6.40)	3.54 (1.72-7.30)
**On temporary or partial on lay-off because of COVID-19**				
No	82	51	1 [reference]	1 [reference]
Yes	49	69	2.26 (1.36-3.76)	2.09 (1.14-3.83)
**Household income change after the pandemic**				

Increased or remained the same	49.3	27.8	1 [reference]	1 [reference]
Decreased	50.7	72.2	2.53 (1.54-4.15)	3.21 (1.80-5.75)
**Falling behind with bills**				
No	76.3	59.7	1 [reference]	1 [reference]
Yes	23.7	40.3	2.18 (1.31-3.62)	1.95 (1.09-3.50)
**Financial difficulties in buying food**				
No	74.3	68.5	1 [reference]	1 [reference]
Yes	25.7	31.5	1.78 (1.08-2.94)	1.29 (0.72-2.30)
**Financial difficulties in buying hygiene products**				
No	77	57.5	1 [reference]	1 [reference]
Yes	23	42.5	2.47 (1.49-4.12)	1.95 (1.10-3.48)
**Financial difficulties to pay phone and internet**				
No	80.1	49.6	1 [reference]	1 [reference]
Yes	19.9	50.4	4.10 (2.42-6.92)	3.02 (1.65-5.53)
Kids go to school for a meal				
No	97.0	90.1	1 [reference]	1 [reference]
Yes	3.0	9.9	3.61 (1.14-11.37)	2.02 (0.57-7.19)
