# Supplementary material for: Cohort profile: Health trajectories of Immigrant Children (CRIAS)–a prospective cohort study in the metropolitan area of Lisbon, Portugal
Source: BMJ Open. 2022 Oct 25;12(10):e061919. doi: 10.1136/bmjopen-2022-061919 (PMC9608527; doi:10.1136/bmjopen-2022-061919)
Supplement: Author's manuscript [file bmjopen-2022-061919.draft_revisions.pdf]

BMJ Open is committed to open peer review. As part of this commitment we make the peer review history of every article we publish publicly available.

When an article is published we post the peer reviewers' comments and the authors' responses online. We also post the versions of the paper that were used during peer review. These are the versions that the peer review comments apply to.

The versions of the paper that follow are the versions that were submitted during the peer review process. They are not the versions of record or the final published versions. They should not be cited or distributed as the published version of this manuscript.

BMJ Open is an open access journal and the full, final, typeset and author-corrected version of record of the manuscript is available on our site with no access controls, subscription charges or pay-per-view fees (http://bmjopen.bmj.com).

If you have any questions on BMJ Open's open peer review process please email info.bmjopen@bmj.com

BMJ Open

COHORT PROFILE: HEALTH TRAJECTORIES OF IMMIGRANT CHILDREN – CRIAS, A PROSPECTIVE COHORT STUDY IN THE METROPOLITAN AREA OF LISBON, PORTUGAL

Journal:	*BMJ Open*
Manuscript ID	bmjopen-2022-061919
Article Type:	Cohort profile
Date Submitted by the Author:	10-Feb-2022

Complete List of Authors:	Muggli, Zelia; Universidade Nova de Lisboa Instituto de Higiene e Medicina Tropical,<br>Mertens, Thierry; Universidade Nova de Lisboa Instituto de Higiene e Medicina Tropical<br>Amado, Regina; Universidade Nova de Lisboa Instituto de Higiene e Medicina Tropical<br>Teixeira, A. L.; Universidade Nova de Lisboa Faculdade de Ciências Sociais e Humanas, CICS.NOVA - NOVA FCSH<br>Vaz, Dora; ARSLVT, ACES Amadora,Unidade de Saúde Pública<br>Pires, Melanie; ARSLVT, ACES Amadora , USF Ribeiro Sanches<br>Loureiro, Helena ; Hospital Amadora-Sintra, Paediatrics<br>Fronteira, Inês; Universidade Nova de Lisboa Instituto de Higiene e Medicina Tropical<br>Abecassis, Ana; Universidade Nova de Lisboa Instituto de Higiene e Medicina Tropical<br>Silva, António ; ARSLVT, Saúde Pública<br>Martins , MDRO; Universidade Nova de Lisboa, Instituto de Higiene e Medicina Tropical

Keywords:	EPIDEMIOLOGY, Health policy < HEALTH SERVICES ADMINISTRATION & MANAGEMENT, Organisation of health services < HEALTH SERVICES ADMINISTRATION & MANAGEMENT, Community child health < PAEDIATRICS

SCHOLARONE™
Manuscripts

I, the Submitting Author has the right to grant and does grant on behalf of all authors of the Work (as defined in the below author licence), an exclusive licence and/or a non-exclusive licence for contributions from authors who are: i) UK Crown employees; ii) where BMJ has agreed a CC-BY licence shall apply, and/or iii) in accordance with the terms applicable for US Federal Government officers or employees acting as part of their official duties; on a worldwide, perpetual, irrevocable, royalty-free basis to BMJ Publishing Group Ltd ("BMJ") its licensees and where the relevant Journal is co-owned by BMJ to the co-owners of the Journal, to publish the Work in this journal and any other BMJ products and to exploit all rights, as set out in our licence.

The Submitting Author accepts and understands that any supply made under these terms is made by BMJ to the Submitting Author unless you are acting as an employee on behalf of your employer or a postgraduate student of an affiliated institution which is paying any applicable article publishing charge ("APC") for Open Access articles. Where the Submitting Author wishes to make the Work available on an Open Access basis (and intends to pay the relevant APC), the terms of reuse of such Open Access shall be governed by a Creative Commons licence – details of these licences and which Creative Commons licence will apply to this Work are set out in our licence referred to above.

Other than as permitted in any relevant BMJ Author's Self Archiving Policies, I confirm this Work has not been accepted for publication elsewhere, is not being considered for publication elsewhere and does not duplicate material already published. I confirm all authors consent to publication of this Work and authorise the granting of this licence.

For peer review only

COHORT PROFILE: HEALTH TRAJECTORIES OF IMMIGRANT CHILDREN – CRIAS, A PROSPECTIVE COHORT STUDY IN THE METROPOLITAN AREA OF LISBON, PORTUGAL

Zélia Muggli[1], Thierry Mertens[1], Regina Amado[1], Ana L. Teixeira[2] Dora Vaz[3], Melanie Pires[3], Helena Loureiro[4], Inês Fronteira[1], Ana Abecassis[1], António Carlos Silva[5,6] Maria Rosário O. Martins[1]

Correspondence to Prof Maria Rosário O. Martins; mrfom@ihmt.unl.pt

[1] Global Health and Tropical Medicine, Institute of Hygiene and Tropical Medicine, NOVA University, Lisbon, Portugal

[2] Interdisciplinary Centre of Social Sciences (CICS.NOVA), Faculty of Social Sciences and Humanities (NOVA FCSH), Lisbon, Portugal

[3] Amadora Primary Care Health Centres Group, Regional Health Administration of Lisbon and Tagus Valley, Amadora, Portugal

[4] Department of Paediatrics, Hospital Prof. Dr. Fernando Fonseca, Amadora , Portugal

[5] Public Health Department, Regional Health Administration of Lisbon and Tagus Valley, Lisbon, Portugal

[6] AJPAS, NGO, Associação de Intervenção Comunitária, Desenvolvimento Social e de Saúde, Amadora, Portugal

For peer review only

Word count : 3971

For peer review only - http://bmjopen.bmj.com/site/about/guidelines.xhtml

ABSTRACT

Purpose: CRIAS is a prospective cohort study created to better understand the health trajectories of immigrant and native children in the Lisbon area, Portugal. It aims to analyse child health determinants, focussing on migration, and identify factors associated with physical, cognitive and social-emotional development outcomes and utilization of health services.

Participants: The original CRIAS was set up to include 604 children born in 2015, of which 50% immigrant, and their parents. We recruited 420 children between June 2019 and March 2020. Data was collected at age 4 and 5 years; follow-up at age 6/7 is under way.

Findings to date: Baseline data at age 4 showed immigrant children to be at socioeconomic disadvantage . Utilisation of primary care was higher for native children (78.2% vs 73.9%), while utilisation of the hospital emergency department was higher for immigrants (53.2% vs 40.6%). More immigrant children had psychomotor development test items to monitor (38.5% vs 28.3%); being a 1st generation immigrant child increased the chance (aOR 2.2; 95%CI: 1.06-4.76) of emotional and behavioural difficulties. At age 5 follow-up (1st year COVID-19 pandemic), inequalities in primary care utilization increased (80.2% native vs 70.2% immigrant); emergency room use dropped significantly (-45% for immigrant vs -32% for natives); psychomotor development continued to require more monitoring among immigrant children (33.9% vs 21.6%). Socioeconomic inequalities exacerbated: due to the COVID-19 pandemic, immigrant parents were 3.5 times more likely to be unemployed and 3.2 times to have their household income decreased.

Future plans: To contact families in January 2022 for key outcomes follow-up and socioeconomic information update; 2nd wave of data on emotional/behaviour problems at age 6-7. Additional funds awarded by the National Science Foundation will allow 900 more

For peer review only - http://bmjopen.bmj.com/site/about/guidelines.xhtml

children from 4 other Lisbon Area Municipalities to be included in the cohort from February 2022 (cohort-sequential design).

Strengths and limitations of this study

- **First cohort study in the Lisbon Area, Portugal adding valuable data about the little-known health trajectories of immigrant children, with the potential to identify early interventions**
- **Strategic partnership between the University, National Health Service and an NGO, generating a unique repository linking data from health centres, hospital and face-to-face questionnaires collected over time.**
- **Allowed follow-up of the more vulnerable families during the COVID-19 pandemic**
- **A limitation was the conclusion of recruitment 3 months before planned due to the COVID-19 pandemic restrictions. This context has also been a key challenge to the follow-up.**
- **Absence of information on children who don't attend primary health care is another limitation**

For peer review only - http://bmjopen.bmj.com/site/about/guidelines.xhtml

INTRODUCTION

A rapidly growing part of the population in Europe is composed of immigrants. In 2020, Portugal registered its highest number:6.4% of the country's population were immigrants mostly non-EU nationals (69%); Brazil (28%) and Portuguese speaking countries in Africa are the main countries of origin, but an increasingly significant number arrived from Asia [1]. The health effects of the migration process are complex and the need to increase the knowledge base, especially for more vulnerable groups like children, has been highlighted at the national and international levels [2–4].

Immigrant children often live in low income and socially disadvantaged environments which can adversely impact their health outcomes [5,6]. Previous studies show that immigrant children present distinct health needs and more frequent health problems [7], including being more at risk of overweight and obesity and some infectious diseases [8,9]. Inequalities in access and utilization of healthcare services were observed with immigrant children having less probability of having a regular healthcare provider and using dental services but using more hospital emergency departments [10] The findings on vaccination [11–13] and mental health difficulties [14–18] tend to be less favourable in immigrant children. However, results are not always consistent – this might reflect the heterogenicity of the immigrant groups between countries or even among regions. Hence the importance of conducting research at a more regional level within a single country. In Portugal there is an overall lack of studies on migrant child health.

Childhood, especially the first 8 years, encompasses a rapid period of growth and development which plays a key role for health and wellbeing across the life course [19]. This period is highly influenced by the environment where the child grows and develops, namely by socioeconomic factors [20].Therefore, gaining evidence on children's health and development profiles, during

For peer review only

the pre-school period and following them in a longitudinal study offers the possibility to formulate and implement early interventions. These can help children not only to reach their full potential when starting school, but also to have a positive impact on their future health, wellbeing and educational outcomes.

The CRIAS cohort is the first longitudinal study in the Metropolitan Area of Lisbon, Portugal, focussing on gaining a better understanding on the health and development trajectories of immigrant and native children over time, given their respective socioeconomic and cultural contexts. The aim of the CRIAS cohort is to explore the effects of exposures, focussing on migration, and identify risk factors associated with the physical, cognitive and social-emotional development outcomes, as well as with access and utilization of healthcare services. It addresses at the same time the scarcity of data in the country about migrant children health, in particular cohort data, and the need to have evidence-based information on contextual factors driving inequalities in health. This will provide opportunities for the early identification of modifiable risk factors, and to facilitate early implementation of effective interventions towards health equity while fostering a better integration of immigrant families. The development of this cohort arises from a strategic and unique partnership between the University, National Health Service and AJPAS- a local NGO focussing on the needs of immigrant populations.

Children born in 2015, residing in the Metropolitan Lisbon Region- Amadora, are followed up and findings compared between immigrants and natives. We defined as immigrant, a child residing in Portugal and born in a non-EU country (1st generation immigrant) or having one parent born in a non-EU country; native children are born in Portugal with both parents born in Portugal. Measurements were scheduled at the ages of 4, 5, 6/7 and further assessments are planned for as long as possible, according to the key ages of the National Child and Youth Health Programme [21].

For peer review only - http://bmjopen.bmj.com/site/about/guidelines.xhtml

COHORT DESCRIPTION

Setting

The CRIAS study is conducted in the Amadora municipality, in the Metropolitan Area of Lisbon, Portugal. With 171,500 inhabitants in 2021 [22] it is the most densely populated municipality in the country. With a history of immigrants settlement , 13% of its population had a foreign nationality in 2020, making Amadora the second municipality in Portugal,with the highest density of foreign residents – 977/km^2 [1,23]. It is served by 10 Primary Health Care Centres (9 up to December 2020), from now on referred as health centres, and 1 referral hospital –Hospital Prof. Doutor Fernando Fonseca (HFF).

Recruitment and participants

Recruitment was scheduled to take place in the 9 health centres between June 2019-June 2020. However, due to the COVID-19 pandemic, it was interrupted in March 2020. To be included in the study, children had to be born in 2015 and to have records of attending the health centre in the previous 2 years. There were 1009 children with these characteristics. Based on a previous study [24] we assumed that around 30% of users are immigrant children i.e.302. In order to compare outcomes between immigrant and native children we paired 1:1, resulting in a total of 604 children eligible to participate, together with parents/caregivers.

Families were enrolled while in attendance at the health centre. Recruitment weeks were randomly distributed among the 9 health centres and the number of children recruited was proportional to the number registered in each centre. During recruitment, 499 parents/caregivers were approached; the participation rate was 84%. From the 420 children enrolled, 217 are immigrant (51,6%) and 203 native, 6 children are twins (4 immigrant, 2 native).

For peer review only

At age 5 follow-up, children's health centre records showed that 7 children have moved to another municipality. Figure 1 illustrates cohort participation

Starting in January 2022, families are being contacted by phone to arrange further follow-up assessments; loss to follow-up rates can then be better evaluated .

Figure 1. Flow diagram of CRIAS cohort participants.

In order to facilitate enrolment and minimize loss to follow up several strategies were implemented:

- A pilot study (n=33) was conducted to verify acceptability of the questionnaires in terms of content and time by the families
- Active engagement with health professionals. An official presentation of the study to all staff took place in a public venue, followed by further kick-off meetings in each health centre where a interlocutor was nominated to interface with the study team. Health professionals are part of the research team.
- An international team of 6 researchers from 5 different Portuguese speaking countries, and proficient in 6 different languages, was trained to carry out recruitment and conduct initial interviews.
- All participants received details on the study objectives and direct contacts of the research principal investigator. Confidentiality issues and other questions raised were addressed in a prompt and culturally sensitive manner by the researchers. Interviews were conducted in total privacy in specially allocated rooms.
- Communication with parents is kept by phone each year and the contacts database is updated regularly with information from the health centres. Feedback (post/email) on screening outcomes is provided and when needed direction to further assessments is given. Face-to-face contact is preferred whenever possible.
- A local NGO -AJPAS working with the immigrant community is involved to facilitate

For peer review only

participation

Data collection

The first wave of data collection at age 4 was carried out in the health centres between June 2019 and March 2020 by a team of 6 researchers using structured questionnaires. All interviewers received the same detailed information and training on the interview process. Feedback on the information collected was routinely given and inconsistencies were corrected when needed by senior researchers in the team .

Face-to-face interviews were held with parents/caregivers and as a first step we collected family's socioeconomic and demographic characteristics migration history and child health information. The interviews were conducted mostly in Portuguese with other languages used when needed (e.g. Creole, English). This was followed by a self-administered screening questionnaire on the child's emotional and behavioural difficulties – the Strengths and Difficulties Questionnaire (SDQ), available in validated translations and administered in the preferred language of the participant.

By June 2020, 85% of all COVID-19 cases in the country were in the Metropolitan Area of Lisbon, Amadora being one of the most affected municipalities [25]. To explore the socioeconomic dynamics of the cohort families during the Covid 19 pandemic, an intermediate data collection was undertaken in July 2020. Phone interviews were conducted applying a semi-structured questionnaire exploring changes in employment and household income, material deprivation and difficulties related to the lockdown including access to health care.

The restriction measures adopted during the COVID-19 pandemic have limited the access to health centres and delayed collection of follow-up data, especially if involving face-to face contact. Nevertheless, baseline (children aged 4) and first follow-up (children aged 5) clinical data from electronic records for primary care and hospital emergency department visits were collected from November 2019 to October 2021

For peer review only

Figure 2 includes a schematic representation of data collection.

Instruments and measures

Parents/caregivers were interviewed using a pilot-tested structured questionnaire to collect: 1) Information on parents – sex; age; country of birth; education based on the International Standard Classification of Education [26] categorized in 4 levels– lower education level (no schooling, primary education), 9 years of schooling completed, secondary education, university degree; occupation was classified as per the Portuguese Classification of Professions and summarized in 4 skill levels –high skilled occupations (managers, professionals, legislatives, specialized technicians), medium skills (clerks, personal service workers industrial workers, machine operators), low-skilled (unqualified workers) and no defined occupation, according to the International Standard Classification of Occupations [27]; household monthly income and employment status; out-of-pocket expenses and private health insurance; for immigrants– reasons for emigration and length of stay in Portugal. 2) Sociodemographic information on children – sex, country of birth, language spoken at home, length of stay in Portugal for immigrant children. 3) Child health history – perceived health of the child by the parents over the last 12 months measured in a 5-point Likert scale and summarized in 3 groups; gestational age (< 37weeks; ≥ 37 weeks), type of delivery (vaginal; caesarean); total and exclusive duration of breastfeeding, fruit and vegetables intake according to recommend servings/day by the Portuguese Health Directorate (2 or more servings of fruits and 3 or more serving of vegetables a day) [28]; number of illness episodes in the last 3 months and symptoms 3) Child environment variables – family structure (single-parent, both parents, extended family); household size defined as large when 5 or more people, ratio people/number of bedrooms; childcare arrangements (pre-school, staying at home or others).

A second questionnaire was self-administered to one parent or main caregiver – the Strengths and Difficulties Questionnaire (SDQ) [29], parents version for 4-17 years old, translated to

For peer review only - http://bmjopen.bmj.com/site/about/guidelines.xhtml

Portuguese by Fleitlich and Loureiro [30], as well as in validated translations in other languages. The SDQ is a brief questionnaire to assess child emotional and behavioural difficulties. It has been widely used and validated in various research, clinical, and community settings, including in multi-ethnic populations of children [31,32] and in several countries including Portugal [33]. Consists of 5 subscales, 4 measuring difficulties, which can be grouped into in 2 broad categories of behaviours: externalizing (conduct problems + hyperactivity) and internalizing (emotional + peer problems) behaviours. One subscale measures a strength – prosocial behaviour. A total score of difficulties can also be calculated to classify results as normal, borderline or at risk. Children scoring borderline or at risk were advised to repeat the SDQ 6 months after the first test, with those classified as at risk being referred to their doctor

Physical health and development information for the ages of 4 and 5 years old was collected by two medical doctors who are part of the team from the medical records available on the SCLINICO primary health care information system. Measurements were performed during the routine health assessments by health professionals in line with the National Child and Youth Health Programme guidelines. Anthropometric measures were recorded and underweight, overweight and obesity were classified using the World Health Organization Child Growth Standards charts [34]. Visual acuity was verified using the Snellen E-Chart with optimal vision reported as 10/10 and changes in ocular alignment were inspected. Dental health was evaluated for the presence of caries (yes/no). Information on the completion (yes/no) of the vaccination schedule was collected from the records as well.

Psychomotor development was evaluated by the modified Mary Sheridan screening test, assessed during routine child health visits at 4 and 5 years of age [21] . The test covers 4 domains – posture and gross motor skills, vision and fine motor skills, hearing and language and social adaptation. Outcomes of the test were categorized in “normal” if all items were fulfilled and in “to monitor” if one or more items in one or a combination of domains was not

For peer review only

For peer review only - http://bmjopen.bmj.com/site/about/guidelines.xhtml

achieved.

Additional variables from electronic medical records were related to access and utilization of healthcare services – allocation of a family doctor, number of consultations received in a one year period (2019 and 2020), including diagnosis based on the International Classification of Primary Care (ICPC-2) clinical activity for the health centres; for hospital emergency room – annual visits (yes/no and frequent users ≥ 4 visits/year) and characteristics of visits such as time of the day, diagnosis (ICD-9-CM) and clinical priority (Manchester Triage Scale). Data on utilization of the emergency department was provided by the hospital.

Figure 2. Representation of data collection and main measures of the CRIAS cohort study
*Key outcomes

Data linkage

Electronic medical records are managed in primary health care and in the hospital in two different information systems, SCLINICO and SORIAN respectively. We link 3 different datasets: data collected through interviews; primary health care data and hospital data. This linkage is accomplished using the SNS (National Health System) user number. The information is obtained from the electronic medical records with the SNS number which is then returned to the project coordinator who matches this number with the ID code of the child. The key which assigns the SNS user number to the name of the child and to the ID code is kept password protected by the coordinator of the study. The integrated cohort database available for analysis only includes the ID code.

Patient and public involvement

The NGO AJPAS, founded by immigrants and located in Amadora Municipality, and the members of the regional health authorities have been involved in the design, governance and general oversight of all phases of the research to date. Study participants have been encouraged to communicate to the research team by phone and email. Reports and presentations are frequently shared with key stakeholder groups. Members of the NGO AJPAS have been

For peer review only

trained to also participate as interviewers in the survey on the socioeconomic impact of the COVID-19 pandemic providing economic, social and legal support whenever requested by the study participants .

For peer review only

For peer review only - http://bmjopen.bmj.com/site/about/guidelines.xhtml

FINDINGS TO DATE

Baseline characteristics of children and parents/caregivers

A large majority of the 417 parent/caregivers interviewed were women (88%), nearly all were the mothers. The main countries of origin of immigrant parents/caregivers were Cape Verde (n=60), Angola (n=28), Brazil (n=28), and Guinea-Bissau (n=22). The main reasons for immigration given by the mother were family reunification (28.9%), obtaining a better education (27.6%), economic reasons (22.4%), with 3.3% having moved because of war; the median length of stay in years in Portugal was 9 years (min.0.1-max.37). Information on the main sociodemographic characteristics of the families is found in table 1.

Table 1. Baseline characteristics of the parents/caregivers of children in the CRIAS cohort

Parents/caregivers n=417	Immigrant children n (%)	Native children n (%)	Total n (%)	p Value
Women	187 (86.6)	179 (89.1)	366 (87.8)	0.440*
Men	29 (13.4)	22 (10.9)	51 (12.2)	
**Age n=417**				0.213**
Median (min-max; IQR)	34 (20-75;10)	35 (18-68;10)	35(18-75;10)	
**Relationship with child n=417**				0.262*
Mother	182 (84.3)	177 (88.1)	359 (86.1)	
Others	34 (15.7)	24 (11.9)	58 (13.9)	
**Educational level n=416**				0.115*
Lower education	40 (18.6)	27 (13.4)	67 (16.1)	
9 years completed	41 (19.1)	45 (22.4)	86 (20.7)	
Secondary education	91 (42.3)	73 (36.3)	164 (39.4)	
University degree	43 (20.0)	56 (27.9)	99 (23.8)	
**Occupation n= 414**				<0.001*
High skilled	34 (16.0)	69 (34.3)	103 (24.9)	
Medium skilled	99 (46.5)	102 (50.7)	201 (48.6)	
Low skilled	75 (35.2)	20 (10.0)	95 (22.9)	
Non- defined	5 (2.3)	10 (5.0)	15 (3.6)	

For peer review only - http://bmjopen.bmj.com/site/about/guidelines.xhtml

**Employment status n=417**				0.009*
Employed with a contract	135 (62.5)	157 (78.1)	292 (70.0)	
Employed without a contract	20 (9.3)	5 (2.5)	25 (6.0)	
Unemployed with benefits	13 (6.0)	10 (5.0)	23 (5.5)	
Unemployed without benefits	18 (8.3)	11(5.5)	29 (7.0)	
Self-employed	16 (7.4)	9 (4.5)	25 (6.0)	
Other [a]	14 (6.5)	9 (4.5)	23 (5.5)	
**Household monthly income n=395**				<0.001*
<500 €	39 (19.1)	12 (6.3)	51 (12.9)	
>500—750	66 (32.4)	44 (23.0)	110 (27.8)	
>750—1000€	38 (18.6)	34 (17.8)	72 (18.2)	
>1000—1500€	36 (17.6)	43 (22.5)	79 (20.0)	
>1500—2000€	16 (7.8)	24 (12.6)	40 (10.1)	
>2000€	9 (4.4)	34 (17.8)	43 (10.9)	

Significance level 5%. *Pearson- Chi square statistical test ** Mann-Whitney U statistical test

[a] students, stay-at-home parents, retired

Higher education degrees were more frequent in parents of native children whereas non-qualified low skilled workers and the unemployed were mostly from families of immigrant background. The distribution of family income was unequal, with more than three times (17.6% versus 4.4%) the families of native children declaring an income above 2000€/month; in contrast close to three times (18.5% versus 6.7%) more immigrant families declared an income of less than 500€/month.More immigrant children grow in single-parent families (19.4% vs 13.8%) and large households (36.2% vs 28.2%)

Information collected on the 203 native and 217 (51,6%) immigrant children in the cohort , showed that 41 children were born in a non-EU country. They originated mainly from the Community of Portuguese Speaking Countries: 13 from Brazil, 8 from Angola, followed by Guinea-Bissau with 6 and Cape Verde with 4. The median length of stay in Portugal of these 1st generation immigrant children was 18 months (1 min.- 48 max.). Children from the more recent immigrant communities from countries such as India, Nepal or Eritrea, are also present

For peer review only - http://bmjopen.bmj.com/site/about/guidelines.xhtml

in the study. The only language spoken in 268 households (64%) was Portuguese while 17 other languages were spoken, ranging from Nepalese to Mandarin and Tigrinya. After the Portuguese, the most common language spoken was a combination of Creole and Portuguese (20%), spoken not only by immigrant families but also in 6.4% of households of native children. Table 2 shows other relevant characteristics of participating children

Table 2 Main characteristics of children in the CRIAS cohort at baseline

Characteristics of the children	Immigrant n (%)	Native n (%)	Total n (%)	p Value
	217 (51.7)	203 (48.3)	420 (100)	
**Gender n=420**				0.689*
Girls	109 (50.2)	98 (48.3)	207 (49.3)	
Boys	108 (49.8)	105 (51.7)	213 (50.7)	
**Gestational age n=413**				0.463*
<37weeks – Preterm	15 (7.0)	18 (9.0)	33 (8.0)	
>37 weeks	198 (93)	182 (91)	380 (92)	
**Birthweight n=385**				0.531*
<2500g– Low Birth Weight	19 (9.9)	16 (8.1)	35 (9.0)	
>2500g	172 (90.1)	181 (91.9)	353 (91.0)	
**Breastfeeding n=419**				0.152*
Yes	203 (93.1)	179 (89.1)	382 (91.2)	
No	15 (6.9)	22 (10.9)	37 (8.8)	
**Total Duration of breast feeding n=375**				<0.001**
Median months (min-max; IQR)	12 (0-53;18)	6 (0-48;14)	10 (0-53;15)	
**Family structure n=419**				0.084*
Both parents	99 (45.8)	117 (57.6)	216 (51.6)	
Both parents and others	30 (13.9)	27 (13.3)	57 (13.6)	
Single-parent families	42 (19.4)	28 (13.8)	70 (16.7)	
One parent and others/others	45 (20.9)	31 (15.3)	76 (18.1)	
**Large households (>=5 people) n=420**				0.079*
Yes	79 (36.2)	57 (28.2)	136 (32.4)	
No	139 (63.8)	145 (71.8)	284 (67.6)	
**Ratio people in household/ number bedrooms n=420**				<0.001***
Mean (95% CI)	2.00 (1.89 to 2.11)	1.73 (1.65 to 1.81)	1.87 (1.80 to 1.94)	
**Childcare arrangements n=417**				0.329*

For peer review only - http://bmjopen.bmj.com/site/about/guidelines.xhtml

State pre-school	83 (38.4)	81 (39.9)	164 (39.1)	
Private pre-school	94 (43.5)	98 (48.3)	192(45.8)	
Stays home w/mother	15 (6.9)	8 (3.9)	23 (5.5)	
Other	24 (11.1)	16 (7.9)	40 (9.5)	
**Assigned family doctor n=420**				<0.001*
Yes	161(73.9)	179 (88.6)	340 (81.0)	
No	57 (26.1)	23 (11.4)	80 (19.0)	
**Health coverage beyond National Health Service n=417**				
Yes	63 (29.3)	104 (51.5)	167 (40.0)	<0.001*
No	152 (70.7)	98 (48.5)	250 (60.0)	

Significance level 5%. *Pearson- Chi square statistical test ** Mann-Whitney U statistical test

[a] students, stay-at-home parents, retired

Findings on key outcomes and other variables (at ages 4 and 5)

At baseline (age 4) the perceived health of the child was considered to be very good or good by 80% of the parents. The median number of parent reported episodes of illness in the last 3 months was 1 and did not differ significantly between immigrant and native children. Most frequently reported complaints, for immigrant and native children respectively, were related with the respiratory tract 49.5% vs 66.5%, fever 18.8% vs 17.3 %, skin problems 6.4% vs 4% and digestive complains 7.9% vs 6.5%. Vaccination rates were above 90% for all children. In the Modified Mary Sheridan test to evaluate psychomotor development, more immigrant children were found to have items to monitor (38%vs 28%), with a similar number (25%) of native and immigrant children having not achieved items in the vision and fine motor skills domain. The above information is shown on supplementary table 1. The findings on emotional and behavioural difficulties suggest that a low family income (aOR 4.5; 95%CI: 1.43-13.95), low parental education level (aOR 2.5; 95%CI: 1.11- 5.16) and being a 1st generation immigrant child (aOR 2.2; 95%CI: 1.06-4.76) increased significantly the chance of developing emotional and behavioural difficulties [35].

Main variables collected on the utilization of health services are in table 3. Over a quarter (26%) of immigrant children did not have a regular allocated family doctor and 36% of 1st

For peer review only

For peer review only - http://bmjopen.bmj.com/site/about/guidelines.xhtml

generation immigrants did not receive the 4 years-old routine health assessments. Immigrant children were those who used less primary care (74% versus 78%), but used most the hospital emergency rooms (53.2% versus 40.6%). Most frequent diagnosis on primary care visits were respiratory, digestive, skin and anaemias, with more immigrant children being diagnosed with atopic dermatitis (14.2% *vs* 6.9%) and anaemias (13.8% *vs* 7.9%).

Other findings at age 4 (supplementary table 2) included dental caries observed in 23% of the children , and a similar number had vision acuity or eyes alignment difficulties with no differences among groups. The recommended intake of fruits and vegetables is not achieved by most children, particularly immigrant children. Overweight was found in 25% of the children (22% in immigrant vs 28% in native children), 6% of children were obese and from a total of 8% underweight children, 72% were immigrant .

The main results from the additional module (July 2020) on the socioeconomic effects of COVID-19 pandemic on families participating in the CRIAS-cohort study are shown on supplementary table 3. Immigrants were more likely to be unemployed due to the COVID-19 pandemic (aOR 3.54, 95% CI 1.72-7.30), more likely to be subject to temporary lay-offs (aOR 2.10, 95% 1.17-3.76) and to have their household income decreased (aOR 3.21, 95% CI 1.80-5.75). Additionally, immigrant families were more likely to fall behind with paying bills (aOR 1.95, 95% CI 1.09-3.50) during the COVID pandemic, to have financial difficulties in buying hygiene products (aOR 1.95, 95% CI 1.10-3.48) and in paying phone and internet (aOR 3.02, 95% CI 1.65-5.53). Immigrant families were also more likely to benefit from school meals during the 1st lockdown (AOR 2.02, 95% 0.57-7.19)

At age 5 years (during 1st year of COVID-19 pandemic), data suggest inequalities in primary care utilization increased with 10% more of natives attending consultations than immigrants (versus 4% in 2019). Emergency room use dropped significantly during this period, but decreased much more for immigrant children (-45% vs -32% for natives). Primary care

For peer review only

consultations for CRIAS children reduced from a total of 735 in 2019 to 606 in 2020 (-17.5%); respiratory symptoms/diagnosis went from 26.2% in 2019 to 12.6% in 2020, being more frequent among native children (16.3% vs 9.2%) . Table 3 compares health care utilization at ages 4 and 5.

Table 3. Utilization of healthcare services by children in the CRIAS cohort at age 4 and 5

	**1st wave of data collection (age 4 years) n=420**				**2nd wave of data collection (age 5 years) n=420**			
**Outcomes**	**Year 2019**				**Year 2020**			
	**Immigrant** n (%)	**Native** n (%)	**Total** n (%)	**p Value**	**Immigrant** n (%)	**Native** n (%)	**Total** n (%)	**p Value**
**Primary care**	**At least one consultation in 2019**			0.296*	**At least one consultation in 2020**			0.018*
Yes	161 (73.9)	158 (78.2)	319 (76.0)		153 (70.2)	162 (80.2)	315 (75.0)	
No	57 (26.1)	44 (21.8)	101 (24.0)		65 (29.8)	40 (19.8)	105 (25.0)	
**Most frequent symptoms/diagnosis in 2019 consultations[a]**					**Most frequent symptoms/diagnosis in 2020 consultations[b]**			
**Respiratory infections**	52 (23.9)	58 (28.7)	110 (26.2)	0.258*	20 (9.2)	33 (16.3)	53 (12.6)	0.027*
**Skin**								
Parasitic and fungal infections	8 (3.7)	3 (1.5)	11(2.6)	0.161**	7 (3.2)	3 (1.5)	10 (2.4)	0.341**
Atopic dermatitis	31 (14.2)	14 (6.9)	45 (10.7)	0.016*	17 (7.8)	12 (5.9)	29 (6.9)	0.453
**Digestive**								
Gastro-enteritis	11 (5.0)	11 (5.4)	22(5.2)	0.854*	5 (2.3)	5 (2.5)	10 (2.4)	0.903*
**Other**	23 (10.6)	13 (6.4)	36 (8.6)	0.132*	16 (7.3)	16 (5.9)	32 (7.6)	0.822*
**Blood**								
Hereditary and iron deficiency anaemias	30 (13.8)	16 (7.9)	46 (11.0)	0.051*	23 (10.6)	23 (10.6)	46 (11.0)	0.784*
	**4 years old routine health assessment**				**5 years old routine health assessment**			
Yes	161 (73.9)	160 (79.2)	321 (76.4)	0.196*	72 (33.2)	64 (31.5)	136 (32.4)	0.718*
No	57 (26.1) [c]	42 (20.8)	99 (23.6)		145 (66.8) [d]	139 (68.5)	284 (67.6)	
**Hospital**	**Visits to Emergency Department in Year 2019**				**Visits to Emergency Department in Year 2020**			
Yes	116 (53.2)	82 (40.6)	198 (47.1)	0.010*	63 (28.9)	54 (26.7)	117 (27.9)	0.621*
No	102 (46.8)	120 (59.4)	222 (52.9)		155 (71.1)	148 (73.3)	303 (72.1)	

For peer review only

For peer review only - http://bmjopen.bmj.com/site/about/guidelines.xhtml

[a] number of consultations in 2019,n=735; [b] number consultations 2020,n=606

Significance level 5%. *Pearson- Chi square statistical test ; ** Fisher's exact test

[c]36.6% 1st generation immigrant children did not received the routine health assessment for 4 year olds

[d]75.6% 1st generation immigrant children did not received the routine health assessment for 5 year olds

The number of children receiving a developmental assessment at age 5 decreased drastically to 25% in both groups, with immigrant children having again more items requiring monitoring (33.9% versus 21.6%).

The findings on emotional and behavioural difficulties have been published [35] and general findings from the 1st wave of data have been published as an abstract in the European Journal of Public Health [36]. Results from the survey on the socioeconomic impact of COVID-19 on immigrant and natives families were awarded with the Human Rights Gold Medal Prize given in 2020 by the National Assembly of the Republic of Portugal; main findings have been submitted to a scientific journal and are under review.

For peer review only

FUTURE PLANS

We are preparing to resume face to face contacts with families and are already contacting the participants by phone. Selected socioeconomic information will be updated. Considering the recent rise in mental health difficulties in children, related with the COVID-19 pandemic, the follow up SDQ assessment might reveal new developments. An additional module to Study Asthma and Allergies in Childhood will be implemented at age 6/7, using ISAAC Methodology [37]. Information on accessing and using health services by the immigrant children in the study will be complemented by a qualitative study.

The conduct of this study and its societal implications led us to extend the study to another 4 Municipalities in the Lisbon Region with the collection of data on further 900 children (450 immigrant) through a sequential-cohort design, likely to include more immigrants from non lusophone countries. Funded by the National Science Foundation, in partnership with 2 local NGOs (AJPAS and Doctors of the World) and 15 health centres, the extension of the cohort study will start in February 2022

We will continue to disseminate our results in conferences, scientific papers and meetings with local NOGs and policy makers at the Regional level. A book is in preparation for the Migrations Observatory in Portugal.

Although Portugal provides free healthcare for all children including undocumented migrants and repeatedly scores high in migrant integration policies, the Migration Policies Index MIPEX 2020 [38] gives a less favourable score for healthcare. Therefore, we will continue to work to translate our findings into policies and services change to improve access, quality of healthcare provision and contribute to better lives of all children.

STRENGTHS AND LIMITATIONS OF THIS STUDY

For peer review only - http://bmjopen.bmj.com/site/about/guidelines.xhtml

This is the first cohort study in the Lisbon Metropolitan Area that provides the opportunity to add valuable data about the little known health trajectories of immigrant children, developed through a strategic partnership between University, health centres, hospital and NGOs. Three different sources of data are linked to provide a unique database incorporating immigrant and native children´s health utilization, health and development outcomes together with families' socioeconomic data over time. The partnership with a local NGO was crucial in the recruitment and follow-up phase and in providing direct support to immigrant families and their children during COVID-19 times. Timely presentation of the results to primary health care professionals potentiates the identification of early interventions. The start of the project just before the COVID-19 pandemic made it possible to follow the more vulnerable families during the pandemic crisis.

The recruitment of all eligible immigrant children (expected n=302) was not possible due to social distancing restrictions required by the COVID-19 pandemic. Difficulties on re-establishing face to face contact with families and on providing the child routine assessments by health professionals often under constrained time and resources in the pandemic context, have delayed follow-up data collection. Another limitation is the absence of information on children who don't attend primary health care

For peer review only

Authors affiliation:

[1] Global Health and Tropical Medicine, Institute of Hygiene and Tropical Medicine, NOVA University, Lisbon, Portugal

[2] Interdisciplinary Centre of Social Sciences (CICS.NOVA), Faculty of Social Sciences and Humanities (NOVA FCSH), Lisbon, Portugal

[3] Amadora Primary Care Health Centres Group, Regional Health Administration of Lisbon and Tagus Valley, Amadora, Portugal

[4] Department of Paediatrics, Hospital Prof. Dr. Fernando Fonseca, Amadora , Portugal

[5] Public Health Department, Regional Health Administration of Lisbon and Tagus Valley, Lisbon, Portugal

[6] AJPAS, NGO, Associação de Intervenção Comunitária, Desenvolvimento Social e de Saúde, Amadora, Portugal

Acknowledgments: We would like to thank all participant families and health centre's staff. The authors acknowledge all the support received from the Executive Direction and Clinical Advisory Board of Amadora group of health centers and Hospital Prof. Doutor Fernando Fonseca and the NGO AJPAS.

Author Contributions: Conceptualization, MROM, ZM, TM, ALT, DV, ACS, IF, AA; methodology, MROM, ZM, TM, IF; validation, MROM, ZM, RA, MP, HL; formal analysis, MROM, ZM, RA; data analysis, MROM, ZM, RA; writing—original draft preparation, ZM, MROM; writing—review and editing, MROM, ZM, TM, IF, AA, DV, AA, ACS; data collection, ZM, RA, MROM, MP, HL; supervision, MROM; project administration, MROM;

For peer review only

funding acquisition, MROM. All authors have read and agreed to the published version of the manuscript.

Ethics approval: Health Ethic Committee of the Regional Health Administration of Lisbon and Tagus Valley, Portugal (001/CES/INV/2019), including an additional approval for the COVID-19 intermediate study (9-2020/CES/2020). A written information and consent form to participate in the study was signed by the parents ,which included permission to assess data from the child's health centre and hospital medical records

Funding: This research was financed by the Asylum, Integration and Migration Fund (PT/2018/FAMI/350), by the National Science Foundation (RESEARCH4COVID-19-065) and by the Multiannual Financial Framework 2014/20, Global Health and Tropical Medicine (GHTM), Institute of Hygiene and Tropical Medicine (IHMT), NOVA University of Lisbon, Lisbon, Portugal. The extension of the cohort study is financed by the National Science Foundation (PTDC/SAU-SER/4664/2020).

Patient consent for publication: Not required

Conflicts of Interest: The authors declare no conflict of interest. The funders had no role in the design of the study; in the collection, analyses, or interpretation of data; in the writing of the manuscript, or in the decision to publish the results.

Data availability statement. Deidentified participant data are available upon reasonable request from the Coordinator of the study MROM - ORCID ID: 0000-0002-7941-0285

For peer review only

REFERENCES

1. SEF/GEPF. Relatório de Imigração, Fronteiras e Asilo 2020 [Internet]. Oeiras, Portugal: Serviço de Estrangeiros e Fronteiras; 2021 Jun. Available from: https://sefstat.sef.pt/Docs/Rifa2020.pdf

2. Alto Comissariado para as Migrações. Plano estratégico para as migrações (2015-2020). [Internet]. Alto Comissariado para as Migrações , Lisboa; Available from: https://www.acm.gov.pt/documents/10181/222357/PEM_net.pdf/3a515909-7e66-41e8-8179-e3aa5e0c7195

3. Abubakar I, Aldridge RW, Devakumar D, Orcutt M, Burns R, Barreto ML, et al. The UCL–Lancet Commission on Migration and Health: the health of a world on the move. The Lancet [Internet]. 2018 Dec [cited 2019 Mar 14];392(10164):2606–54. Available from: https://linkinghub.elsevier.com/retrieve/pii/S0140673618321147

4. WHO European Region. Report on the health of refugees and migrants in the WHO European Region: no public health without refugee and migrant health (2018) [Internet]. WHO; Available from: https://apps.who.int/iris/bitstream/handle/10665/311347/9789289053846-eng.pdf?sequence=1&isAllowed=y

5. Gualdi-Russo E, Toselli S, Masotti S, Marzouk D, Sundquist K, Sundquist J. Health, growth and psychosocial adaptation of immigrant children. European Journal of Public Health [Internet]. 2014 Aug 1 [cited 2021 Oct 21];24(suppl_1):16–25. Available from: https://doi.org/10.1093/eurpub/cku107

6. Ferrer L, Gaillardin F, Cayuela A, Felt E, Hernando C, Collazos F, et al. Children health determinants in a multi-site cohort of immigrant families in Spain (PELFI Study). European Journal of Public Health [Internet]. 2016 Nov [cited 2021 Nov 13];26(suppl_1). Available from: https://academic.oup.com/eurpub/article-lookup/doi/10.1093/eurpub/ckw175.114

7. Carrasco-Sanz A, Leiva-Gea I, Martin-Alvarez L, Torso S del, Esso D van, Hadjipanayis A, et al. Migrant children's health problems, care needs, and inequalities: European primary care paediatricians' perspective. Child: Care, Health and Development [Internet]. 2018 [cited 2020 Apr 5];44(2):183–7. Available from: https://onlinelibrary.wiley.com/doi/abs/10.1111/cch.12538

8. Hjern A. Health of refugee and migrant children : Technical guidance [Internet]. 2018 [cited 2020 Sep 8]. Available from: http://urn.kb.se/resolve?urn=urn:nbn:se:su:diva-164328

For peer review only

9. Jaeger FN, Hossain M, Kiss L, Zimmerman C. The health of migrant children in Switzerland. Int J Public Health. 2012 Aug;57(4):659–71.

10. Markkula N, Cabieses B, Lehti V, Uphoff E, Astorga S, Stutzin F. Use of health services among international migrant children – a systematic review. Global Health [Internet]. 2018 Dec [cited 2020 Mar 9];14(1):52. Available from: https://globalizationandhealth.biomedcentral.com/articles/10.1186/s12992-018-0370-9

11. Pavlopoulou ID, Michail KA, Samoli E, Tsiftis G, Tsoumakas K. Immunization coverage and predictive factors for complete and age-appropriate vaccination among preschoolers in Athens, Greece: a cross- sectional study. BMC Public Health [Internet]. 2013 Dec [cited 2021 Oct 22];13(1):908. Available from: http://bmcpublichealth.biomedcentral.com/articles/10.1186/1471-2458-13-908

12. Poethko-Müller C, Ellert U, Kuhnert R, Neuhauser H, Schlaud M, Schenk L. Vaccination coverage against measles in German-born and foreign-born children and identification of unvaccinated subgroups in Germany. Vaccine [Internet]. 2009 Apr 28 [cited 2021 Oct 22];27(19):2563–9. Available from: https://www.sciencedirect.com/science/article/pii/S0264410X09002072

13. Riise ØR, Laake I, Bergsaker MAR, Nøkleby H, Haugen IL, Storsæter J. Monitoring of timely and delayed vaccinations: a nation-wide registry-based study of Norwegian children aged < 2 years. BMC Pediatrics [Internet]. 2015 Nov 13 [cited 2021 Oct 22];15(1):180. Available from: https://doi.org/10.1186/s12887-015-0487-4

14. Baauw A, Ritz N. Towards better healthcare for migrant and refugee children in Europe. Eur J Pediatr [Internet]. 2018 Feb [cited 2020 Mar 10];177(2):161–2. Available from: http://link.springer.com/10.1007/s00431-017-3019-4

15. Vollebergh WAM, ten Have M, Dekovic M, Oosterwegel A, Pels T, Veenstra R, et al. Mental health in immigrant children in the Netherlands. Soc Psychiatry Psychiatr Epidemiol. 2005 Jun;40(6):489–96.

16. Goodman A, Patel V, Leon DA. Why do British Indian children have an apparent mental health advantage? J Child Psychol Psychiatry [Internet]. 2010 Oct [cited 2020 Sep 8];51(10):1171–83. Available from: https://www.ncbi.nlm.nih.gov/pmc/articles/PMC3428791/

17. Leth I, Niclasen J, Ryding E, Baroud Y, Esbjørn BH. Psychological difficulties among children and adolescents with ethnic Danish, immigrant or refugee background. Scandinavian Journal of Child and Adolescent Psychiatry and Psychology [Internet]. 2014 Mar 25 [cited 2020 May 9];2(1):29–36. Available from: https://tidsskrift.dk/sjcapp/article/view/15814

18. Belhadj Kouider E, Koglin U, Petermann F. Emotional and behavioral problems in migrant children and adolescents in Europe: a systematic review. Eur Child Adolesc Psychiatry [Internet]. 2014 Jun 1 [cited 2020 Dec 4];23(6):373–91. Available from: https://doi.org/10.1007/s00787-013-0485-8

19. World Health Organization Early Child Development Knowledge Network (WHO ECDKN). Early Child Development: A Powerful Equalizer. Final Report of the Early

For peer review only - http://bmjopen.bmj.com/site/about/guidelines.xhtml

Childhood Development Knowledge Network of the Commission on Social Determinants of Health. Geneva: World Health Organization, 2007.

20. Pillas D, Marmot M, Naicker K, Goldblatt P, Morrison J, Pikhart H. Social inequalities in early childhood health and development: a European-wide systematic review. Pediatr Res. 2014 Nov;76(5):418–24.

21. Programa Nacional de Saúde Infantil e Juvenil [Internet]. [cited 2021 Oct 24]. Available from: https://www.dgs.pt/documentos-e-publicacoes/programa-tipo-de-atuacao-em-saude-infantil-e-juvenil.aspx

22. Statistics Portugal. INE - Plataforma de divulgação dos Censos 2021 – Resultados Provisórios [Internet]. [cited 2022 Jan 6]. Available from: https://ine.pt/scripts/db_censos_2021.html

23. PORDATA - População estrangeira com estatuto legal de residente: total e por algumas nacionalidades [Internet]. [cited 2020 Sep 8]. Available from: https://www.pordata.pt/Municipios/Popula%C3%A7%C3%A3o+estrangeira+com+estatuto+legal+de+residente+total+e+por+algumas+nacionalidades-101

24. Jonas JA. The impact of economic crisis on children's health: analysis of the perceptions of migrant and native families'users of primary health care in Amadora [Internet] [masterThesis]. Instituto de Higiene e Medicina Tropical; 2018 [cited 2021 Oct 23]. Available from: https://run.unl.pt/handle/10362/52853

25. Direção-Geral da Saúde sobre o Novo Coronavírus COVID-19. Ministra da Saúde anuncia medidas específicas para a região de Lisboa [Internet]. COVID-19. [cited 2021 Nov 13]. Available from: https://covid19.min-saude.pt/ministra-da-saude-anuncia-medidas-especificas-para-a-regiao-de-lisboa/

26. UNESCO Institute for Statistics. International standard classification of education: ISCED 2011 [Internet]. Montreal, Quebec: UNESCO Institute for Statistics; 2012 [cited 2021 Oct 24]. Available from: http://www.uis.unesco.org/Education/Documents/isced-2011-en.pdf

27. International Labour Office. International Standard Classification of Occupations: ISCO-08. ILO, Geneva; 2012.

28. Rêgo C, Lopes C, Durão C, Pinto E, Mansilha H, Pereira da Silva L, et al. Alimentação Saudável dos 0 aos 6 anos – Linhas de Orientação para Profissionais e Educadores [Internet]. Direção-Geral da Saúde. Ministério da Saúde; 2019 [cited 2021 Oct 24]. Available from: https://run.unl.pt/handle/10362/88539

29. SDQ INFO. [cited 2021 Oct 29]. Available from: https://www.sdqinfo.org/

30. Fleitlich, B., Loureiro, M., Fonseca, A., & Gaspar, F. (2005). Questionário de capacidades e dificuldades (SDQ-Por) [Strengths and Difficulties Questionnaire, Portuguese Version]. Retrieved from www.sdqinfo.org. Questionário de capacidades e dificuldades (SDQ-Por) [Strengths and Difficulties Questionnaire, Portuguese Version]. [Internet]. Available from: www.sdqinfo.org

31. Mieloo CL, Bevaart F, Donker MCH, van Oort FVA, Raat H, Jansen W. Validation of the SDQ in a multi-ethnic population of young children. Eur J Public Health [Internet].

For peer review only - http://bmjopen.bmj.com/site/about/guidelines.xhtml

2014 Feb 1 [cited 2020 Apr 9];24(1):26–32. Available from: https://academic.oup.com/eurpub/article/24/1/26/493856

32. Runge RA, Soellner R. Measuring children's emotional and behavioural problems: are SDQ parent reports from native and immigrant parents comparable? Child and Adolescent Psychiatry and Mental Health [Internet]. 2019 Nov 28 [cited 2020 Sep 3];13(1):46. Available from: https://doi.org/10.1186/s13034-019-0306-z

33. Costa PA, Tasker F, Ramos C, Leal I. Psychometric properties of the parent's versions of the SDQ and the PANAS-X in a community sample of Portuguese parents. Clin Child Psychol Psychiatry [Internet]. 2020 Apr [cited 2020 Apr 9];25(2):520–32. Available from: http://journals.sagepub.com/doi/10.1177/1359104519891759

34. WHO child growth standards: length/height-for-age, weight-for-age, weight-for-length, weight-for-height and body mass index-for-age: methods and development [Internet]. [cited 2021 Oct 23]. Available from: https://www.who.int/publications-detail-redirect/924154693X

35. Muggli Z, Mertens T, -Sá S, Amado R, Teixeira AL, Vaz D, et al. Migration as a Determinant in the Development of Children Emotional and Behavior Problems: A Quantitative Study for Lisbon Region, Portugal. Int J Environ Res Public Health [Internet]. 2021 Jan [cited 2021 Oct 24];18(2):375. Available from: https://www.ncbi.nlm.nih.gov/pmc/articles/PMC7825293/

36. Muggli Z, Amado R, Oliveira Martins MR, Pires M, Vaz D. The health of immigrant children: a cohort study in the Metropolitan area of Lisbon - Portugal. European Journal of Public Health [Internet]. 2020 Sep 1 [cited 2022 Jan 9];30(Supplement_5):ckaa166.787. Available from: https://doi.org/10.1093/eurpub/ckaa166.787

37. Ellwood P, ISAAC Steering Committee, ISAAC Phase Three Study Group, ISAAC International Data Centre. ISAAC: International Study of Asthma and Allergies in Childhood : phase three manual. Auckland, N.Z.: ISAAC International Data Centre; 2000.

38. Portugal | MIPEX 2020 [Internet]. www.mipex.eu. [cited 2021 Oct 24]. Available from: https://www.mipex.eu/portugal

For peer review only

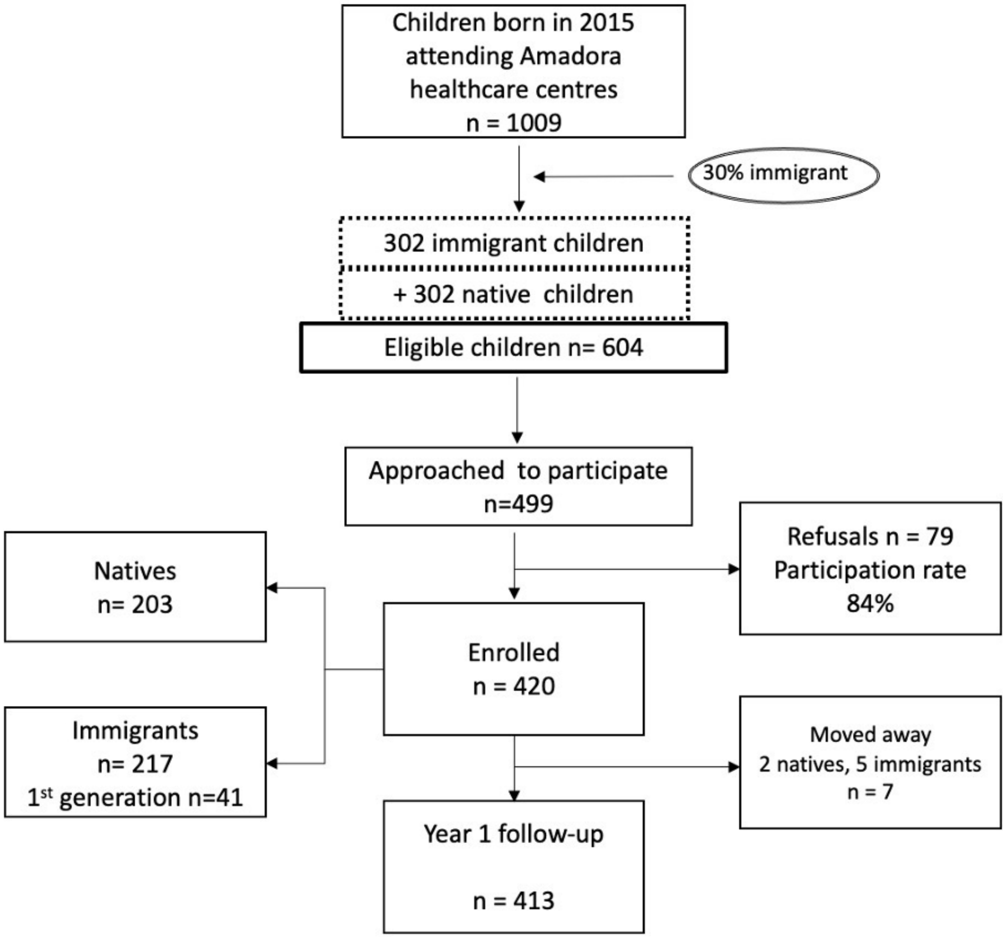

Figure 1. Flow diagram of CRIAS cohort participants.

159x149mm (144 x 144 DPI)

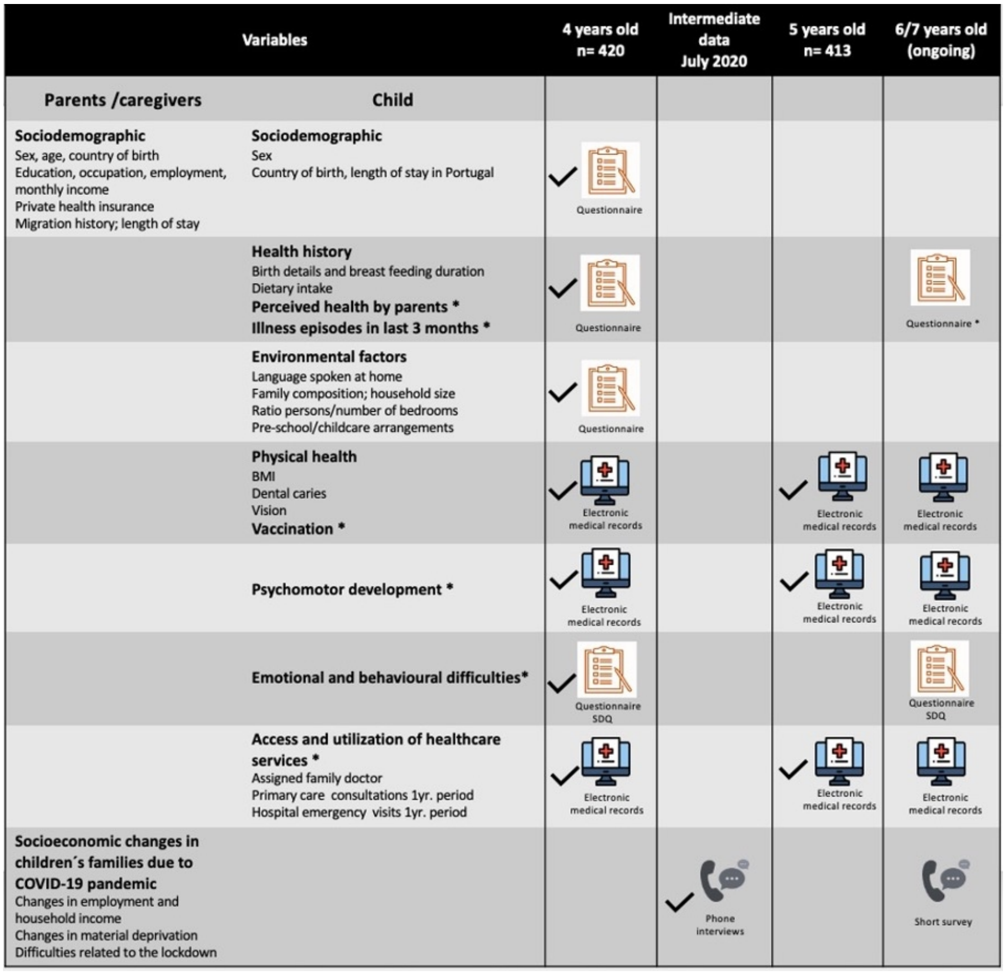

Variables: Parents /caregivers	Variables: Child	4 years old n= 420	Intermediate data July 2020	5 years old n= 413	6/7 years old (ongoing)
**Sociodemographic**<br>Sex, age, country of birth<br>Education, occupation, employment, monthly income<br>Private health insurance<br>Migration history; length of stay	**Sociodemographic**<br>Sex<br>Country of birth, length of stay in Portugal	✓ Questionnaire			
	**Health history**<br>Birth details and breast feeding duration<br>Dietary intake<br>**Perceived health by parents ***<br>**Illness episodes in last 3 months ***	✓ Questionnaire			Questionnaire *
	**Environmental factors**<br>Language spoken at home<br>Family composition; household size<br>Ratio persons/number of bedrooms<br>Pre-school/childcare arrangements	✓ Questionnaire			
	**Physical health**<br>BMI<br>Dental caries<br>Vision<br>**Vaccination ***	✓ Electronic medical records		✓ Electronic medical records	Electronic medical records
	**Psychomotor development ***	✓ Electronic medical records		✓ Electronic medical records	Electronic medical records
	**Emotional and behavioural difficulties***	✓ Questionnaire SDQ			Questionnaire SDQ
	**Access and utilization of healthcare services ***<br>Assigned family doctor<br>Primary care consultations 1yr. period<br>Hospital emergency visits 1yr. period	✓ Electronic medical records		✓ Electronic medical records	Electronic medical records
**Socioeconomic changes in children´s families due to COVID-19 pandemic**<br>Changes in employment and household income<br>Changes in material deprivation<br>Difficulties related to the lockdown			✓ Phone interviews		Short survey

Figure 2. Representation of data collection and main measures of the CRIAS cohort study

159x154mm (144 x 144 DPI)

Supplementary table 1a.Key health oucomes for children at age 4 years

**Key outcomes**	**Immigrant**	**Native**	**Total**	**p Value**
	n (%)	n (%)	n (%)	
	217 (51.7)	203 (48.3)	420 (100)	
**Perceived health by parents** n=415				0.986*
Very good and good	173 (80.1)	160 (80.4)	333 (80.2)	
Fair	39 (18.1)	35 (17.6)	74 (17.8)	
Bad and very bad	4 (1.8)	4 (2.0)	8 (1.9)	
**Illness episodes in previous 3 months (parent reported)** n=410				0.340**
Median (min-max;IQR)	1 (0-5;1)	1 (0-5;2)	1(0-5;1)	0.077*
**Psychomotor development** n=281				
All items achieved	88 (61.5)	99 (71.7)	187 (66.5)	
To monitor	55 (38.5)	39 (28.3)	94 (33.5)	
**Emotional and behavioural difficulties** n=420				
Median externalizing behaviours (min.-max.; IQR)	7 (0-18; 5)[a]	7.5 (1-20; 5)	7 (0-20; 5)	0.950**
Median internalizing behaviours (min.-max.;IQR)	4 (0-13; 4)	3 (0-15; 3)	4 (0-15;4)	<0.001**
**Vaccination up to date** n=416				0.852*
Yes	195 (91.1)	183 (90.6)	378 (90.9)	
No	19 (8.9)	19 (9.4)	38 (9.1)	

Significance level 5%. *Pearson- Chi square statistical test ** Mann-Whitney U statistical

[a] 1st generation immigrant children =9 (min.1-max.15;IQR5) for externalizing behaviours

[b] 36.6% 1st generation immigrant children did not received the routine health assessment age 4

Supplementary table 1b. Psychomotor development at age 5

**Psychomotor development** n=107	Immigrant	Native	Total	P Value
All items achieved	37 (66.1)	40 (78.4)	77 (72.0)	0.155*
To monitor	19 (33.9)	11 (21.6)	30 (28.0)	

Supplementary table 2. Fruit and vegetable intake and Body Mass Index (BMI) of children in the CRIAS cohort

Variables	Immigrant	Native	Total	p Value
	n (%)	n (%)	n (%)	
	217 (51.7)	203 (48.3)	420 (100)	
**Dietary intake**				
**Servings of fruit per day n=412**				<0.001*
Less than 2 servings/day	76 (36.0)	41 (20.4)	117 (27.9)	
2 or more servings/day	135 (64.0)	60 (79.6)	295 (70.2)	
**Servings of vegetables per day n=413**				<0.001*
Less than 3 servings/day	203 (95.8)	195 (97.0)	398 (94.8)	
3 or more servings/day	9 (4.2)	6 (3.0)	15 (3.6)	
**BMI n=314**				
**Overweight**				0.216*
Yes	35 (22.2)	44 (28.2)	79 (25.2)	
No	112 (71.8)	123 (77.8)	235 (74.8)	
**Obesity**				0.824*
Yes	9 (5.1)	8 (5.7)	17 (5.4)	
No	149 (94.9)	148 (94.3)	297 (94.6)	
**Underweight**				0.044*
Yes	18 (11.4)	8 (5.1)	26 (8.3)	
No	148 (88.6)	140	288 (91.7)	

Significance level 5%. *Pearson- Chi square statistical test

For peer review only

Supplementary table 3. Socioeconomic effects of COVID-19 pandemic on families in the CRIAS-cohort study

	All		Natives		Immigrants		p Value*
	n	%	n	%	n	%	
**Unemployed because of COVID-19**							<0.001
No	236	82.2	137	90.1	99	73.3	
Yes	51	17.8	15	9.9	36	26.7	
**On temporary or partial on lay-off because of COVID-19**							
No	133	53	82	62.6	51	42.5	0.001
Yes	118	47	49	37.4	69	57.5	
NA	6						
**Household income change**							<0.001
Increased or remained the same	112	39.3	75	49.3	37	27.8	
Decreased	173	60.7	77	50.7	96	72.2	
**Falling behind with bills**							0.003
No	196	68.5	116	76.3	80	59.7	
Yes	90	(31.5)	36	23.7	54	40.3	
**financial difficulties in buying food**							0.024
No	196	(68.5)	113	74.3	83	61.9	
Yes	90	(31.5)	39	25.7	51	38.1	
**financial difficulties in buying hygiene products**							<0.001
No	194	(67.8)	117	77.0	77	57.5	
Yes	92	(32.2)	35	23.0	57	42.5	
**financial difficulties to pay phone and internet**							<0.001
No	187	(65.8)	121	80.1	66	49.6	
Yes	97	(34.2)	30	19.9	67	50.4	
**kids go to school for a meal**							0.02
No	170	(66.7)	131	97.0	118	90.1	
Yes	85	(33.3)	4	3.0	13	9.9	

*Pearson- Chi square statistical Significance level 5%

For peer review only

STROBE Statement—Checklist of items that should be included in reports of ***cohort studies***

	Item No	Recommendation	Page No
**Title and abstract**	1	(*a*) Indicate the study's design with a commonly used term in the title or the abstract	1
		(*b*) Provide in the abstract an informative and balanced summary of what was done and what was found	2
**Introduction**			
Background/rationale	2	Explain the scientific background and rationale for the investigation being reported	4
Objectives	3	State specific objectives, including any prespecified hypotheses	5
**Methods**			
Study design	4	Present key elements of study design early in the paper	6
Setting	5	Describe the setting, locations, and relevant dates, including periods of recruitment, exposure, follow-up, and data collection	6
Participants	6	(*a*) Give the eligibility criteria, and the sources and methods of selection of participants. Describe methods of follow-up	
		(*b*) For matched studies, give matching criteria and number of exposed and unexposed	7
Variables	7	Clearly define all outcomes, exposures, predictors, potential confounders, and effect modifiers. Give diagnostic criteria, if applicable	9-11
Data sources/ measurement	8*	For each variable of interest, give sources of data and details of methods of assessment (measurement). Describe comparability of assessment methods if there is more than one group	10
Bias	9	Describe any efforts to address potential sources of bias	7
Study size	10	Explain how the study size was arrived at	6
Quantitative variables	11	Explain how quantitative variables were handled in the analyses. If applicable, describe which groupings were chosen and why	9-11
Statistical methods	12	(*a*) Describe all statistical methods, including those used to control for confounding	
		(*b*) Describe any methods used to examine subgroups and interactions	7
		(*c*) Explain how missing data were addressed	
		(*d*) If applicable, explain how loss to follow-up was addressed	
		(*e*) Describe any sensitivity analyses	
**Results**			
Participants	13*	(a) Report numbers of individuals at each stage of study—eg numbers potentially eligible, examined for eligibility, confirmed eligible, included in the study, completing follow-up, and analysed	7
		(b) Give reasons for non-participation at each stage	
		(c) Consider use of a flow diagram	
Descriptive data	14*	(a) Give characteristics of study participants (eg demographic, clinical, social) and information on exposures and potential confounders	13
		(b) Indicate number of participants with missing data for each variable of interest	
		(c) Summarise follow-up time (eg, average and total amount)	
Outcome data	15*	Report numbers of outcome events or summary measures over time	13-18

For peer review only

Main results	16	(*a*) Give unadjusted estimates and, if applicable, confounder-adjusted estimates and their precision (eg, 95% confidence interval). Make clear which confounders were adjusted for and why they were included<br>(*b*) Report category boundaries when continuous variables were categorized<br>(*c*) If relevant, consider translating estimates of relative risk into absolute risk for a meaningful time period	13-18
Other analyses	17	Report other analyses done—eg analyses of subgroups and interactions, and sensitivity analyses	N/A
**Discussion**			
Key results	18	Summarise key results with reference to study objectives	N/A
Limitations	19	Discuss limitations of the study, taking into account sources of potential bias or imprecision. Discuss both direction and magnitude of any potential bias	19
Interpretation	20	Give a cautious overall interpretation of results considering objectives, limitations, multiplicity of analyses, results from similar studies, and other relevant evidence	N/A
Generalisability	21	Discuss the generalisability (external validity) of the study results	N/A
**Other information**			
Funding	22	Give the source of funding and the role of the funders for the present study and, if applicable, for the original study on which the present article is based	23

*Give information separately for exposed and unexposed groups.

Note: An Explanation and Elaboration article discusses each checklist item and gives methodological background and published examples of transparent reporting. The STROBE checklist is best used in conjunction with this article (freely available on the Web sites of PLoS Medicine at http://www.plosmedicine.org/, Annals of Internal Medicine at http://www.annals.org/, and Epidemiology at http://www.epidem.com/). Information on the STROBE Initiative is available at http://www.strobe-statement.org.

For peer review only

BMJ Open

COHORT PROFILE: HEALTH TRAJECTORIES OF IMMIGRANT CHILDREN – CRIAS, A PROSPECTIVE COHORT STUDY IN THE METROPOLITAN AREA OF LISBON, PORTUGAL

Journal:	*BMJ Open*
Manuscript ID	bmjopen-2022-061919.R1
Article Type:	Cohort profile
Date Submitted by the Author:	27-Jul-2022

Complete List of Authors:	Muggli, Zélia; Universidade Nova de Lisboa Instituto de Higiene e Medicina Tropical,<br>Mertens, Thierry; Universidade Nova de Lisboa Instituto de Higiene e Medicina Tropical<br>Amado, Regina; Universidade Nova de Lisboa Instituto de Higiene e Medicina Tropical<br>Teixeira, Ana Lúcia; Universidade Nova de Lisboa Faculdade de Ciências Sociais e Humanas, CICS.NOVA - NOVA FCSH<br>Vaz, Dora; ARSLVT, ACES Amadora,Unidade de Saúde Pública<br>Pires, Melanie; ARSLVT, ACES Amadora , USF Ribeiro Sanches<br>Loureiro, Helena ; Hospital Amadora-Sintra, Paediatrics<br>Fronteira, Inês; Universidade Nova de Lisboa Instituto de Higiene e Medicina Tropical<br>Abecassis, Ana; Universidade Nova de Lisboa Instituto de Higiene e Medicina Tropical<br>Silva, António ; ARSLVT, Saúde Pública<br>Martins , Maria Rosário; Universidade Nova de Lisboa, Instituto de Higiene e Medicina Tropical

**Primary Subject Heading**:	Public health
Secondary Subject Heading:	Global health
Keywords:	EPIDEMIOLOGY, Health policy < HEALTH SERVICES ADMINISTRATION & MANAGEMENT, Organisation of health services < HEALTH SERVICES ADMINISTRATION & MANAGEMENT, Community child health < PAEDIATRICS

SCHOLARONE™
Manuscripts

I, the Submitting Author has the right to grant and does grant on behalf of all authors of the Work (as defined in the below author licence), an exclusive licence and/or a non-exclusive licence for contributions from authors who are: i) UK Crown employees; ii) where BMJ has agreed a CC-BY licence shall apply, and/or iii) in accordance with the terms applicable for US Federal Government officers or employees acting as part of their official duties; on a worldwide, perpetual, irrevocable, royalty-free basis to BMJ Publishing Group Ltd ("BMJ") its licensees and where the relevant Journal is co-owned by BMJ to the co-owners of the Journal, to publish the Work in this journal and any other BMJ products and to exploit all rights, as set out in our licence.

The Submitting Author accepts and understands that any supply made under these terms is made by BMJ to the Submitting Author unless you are acting as an employee on behalf of your employer or a postgraduate student of an affiliated institution which is paying any applicable article publishing charge ("APC") for Open Access articles. Where the Submitting Author wishes to make the Work available on an Open Access basis (and intends to pay the relevant APC), the terms of reuse of such Open Access shall be governed by a Creative Commons licence – details of these licences and which Creative Commons licence will apply to this Work are set out in our licence referred to above.

Other than as permitted in any relevant BMJ Author's Self Archiving Policies, I confirm this Work has not been accepted for publication elsewhere, is not being considered for publication elsewhere and does not duplicate material already published. I confirm all authors consent to publication of this Work and authorise the granting of this licence.

For peer review only

COHORT PROFILE: HEALTH TRAJECTORIES OF IMMIGRANT CHILDREN – CRIAS, A PROSPECTIVE COHORT STUDY IN THE METROPOLITAN AREA OF LISBON, PORTUGAL

Zélia Muggli[1], Thierry E. Mertens[1], Regina Amado[1], Ana L. Teixeira[2] Dora Vaz[3], Melanie Pires[3], Helena Loureiro[4], Inês Fronteira[1], Ana Abecassis[1], António Carlos Silva[5,6] Maria Rosário O. Martins[1]

Correspondence to Prof Maria Rosário O. Martins; mrfom@ihmt.unl.pt

[1] Global Health and Tropical Medicine, Institute of Hygiene and Tropical Medicine, NOVA University, Lisbon, Portugal

[2] Interdisciplinary Centre of Social Sciences (CICS.NOVA), Faculty of Social Sciences and Humanities (NOVA FCSH), Lisbon, Portugal

[3] Amadora Primary Care Health Centres Group, Regional Health Administration of Lisbon and Tagus Valley, Amadora, Portugal

[4] Department of Paediatrics, Hospital Prof. Dr. Fernando Fonseca, Amadora , Portugal

[5] Public Health Department, Regional Health Administration of Lisbon and Tagus Valley, Lisbon, Portugal

[6] AJPAS, NGO, Associação de Intervenção Comunitária, Desenvolvimento Social e de Saúde, Amadora, Portugal

Word count : 3860

For peer review only

For peer review only - http://bmjopen.bmj.com/site/about/guidelines.xhtml

ABSTRACT

Purpose: The CRIAS (Health trajectories of Immigrant Children in Amadora) cohort study was created to explore whether children exposed to a migratory process experience different health risks over time, including physical health, cognitive, socioemotional and behavioural challenges and different health care utilisation patterns

Participants: The original CRIAS was set up to include 604 children born in 2015, of whom 50% immigrant, and their parents. Recruitment of 420 children took place between June 2019 and March 2020 at age 4/5, with follow-up carried out at age 5/6, at age 6/7 currently under way.

Findings to date: Baseline data at age 4/5 (2019-2020) suggested immigrant children to be more likely to belong to families with less income, compared to non-immigrant children. Being a 1st generation immigrant child increased the odds of emotional and behavioural difficulties (aOR 2.2; 95%CI: 1.06-4.76); more immigrant children required monitoring of items in the psychomotor development test (38.5% vs. 28.3%); The prevalence of primary care utilisation was slightly higher among immigrant children (78.0% vs. 73.8%), yet they received less health monitoring assessments for age 4. Utilisation of the hospital emergency department was higher among immigrants (53.2% vs. 40.6%). Age 5 follow-up (2020-2021) confirmed more immigrant children requiring monitoring of psychomotor development, compared to non-immigrant children (33.9% vs. 21.6%). Economic inequalities exacerbated post COVID-19 pandemic confinement with parents of immigrant children 3.2 times more likely to have their household income decreased.

Future plans: Further follow-up will take place at 8, 10, 12/13 and 15 years of age. Funds awarded by the National Science Foundation will allow 900 more children from 4 other Lisbon Area Municipalities to be included in the cohort (cohort-sequential design).

For peer review only

Strengths and limitations of this study

- **The CRIAS cohort is the first study of children in the Lisbon Area, Portugal providing longitudinal data and insights about the little-known health trajectories of immigrant children in this country, with the potential to identify early interventions**
- **Strategic partnership between the University, National Health Service and an NGO, generating a unique repository linking data from health centers, hospital and face-to-face questionnaires collected over time.**
- **The study allowed the follow-up of vulnerable families during the COVID-19 pandemic in Amadora.**
- **Absence of information on children who don't attend primary health care limits the representativeness of the study to those who attend public primary care.**
- **Recruitment of children stopped 3 months before planned due to COVID-19 pandemic restrictions resulting in a smaller sample size. The pandemic context has also been a key challenge to the the first follow-up.**

INTRODUCTION

For the purpose of the CRIAS cohort, an immigrant child was defined as a child residing in Portugal and born in a non-European Union (EU) country (1st generation immigrant) or having one or both parents born in a non-EU country; a non-immigrant child was born in Portugal to both parents born in Portugal.

In Portugal, 6.4% (662,095) of the population in 2020 was made up by foreign nationals. The majority (69%) are non-EU nationals and due to Portugal's colonial past, originating mostly from Brazil (28%) and Portuguese speaking countries in Africa (14.4%), with an increasing number arriving from Asia [1].

Health effects of migration processes are complex and the need to increase the knowledge

For peer review only - http://bmjopen.bmj.com/site/about/guidelines.xhtml

base, especially for more vulnerable groups like children, has been highlighted at the national and international levels [2–4]. European studies report immigrant children often live in low income and socially disadvantaged environments which can adversely impact their health outcomes [4–6]. In Portugal, poor socioeconomic conditions among immigrants, compared to the non-immigrant population have also been reported [7–9]. Comparison between studies and generalization of research findings can be difficult because of the diversity in the definition and contexts of immigrant children across the EU. However, the general trend suggests that immigrant children present distinct health needs and more frequent health problems [10,11], including being more at risk of overweight, obesity and some infectious diseases [12,13]. Inequalities in access and utilization of healthcare services were observed with immigrant children having less probability of having a regular healthcare provider and using dental services but using more frequently hospital emergency departments compared to non-immigrant children [14]. Lower vaccination coverage [15–17] and emotional and behavioural difficulties [18–21] appear to be more frequent among immigrant children compared to non-immigrant children

Childhood, especially the first 8 years, encompasses a period of rapid growth and development which plays a key role for health and wellbeing throughout the life course [22]. This period is highly influenced by the environment where the child grows and develops, and in particular, by socioeconomic factors [23].Therefore, gaining evidence on children's health and development profiles, during the pre-school period and following them in a longitudinal study provides the possibility to formulate and implement early interventions to reduce inequalities [24,25]. These can help children not only to reach their full potential when starting school, but also to have a positive impact on their own future health, wellbeing and educational trajectories but also potentially impact their offspring through transgenerational effects [26].

The first 3–5 years of life appear to be an opportunity window for ensuring adequate nutrition and physical exercise, for promoting parenting quality, child and parents mental health, social-emotional competencies and language and communication skills which are linked to school readiness and better health later on [24,27]. Studies in the United Kingdom suggest that it is possible to mitigate poor outcomes with adequate family support services and interventions in schools [24]

Several birth cohorts where data on immigration status or ethnicity has been collected have

For peer review only

been established in Europe. Whereas some have compared health risks and outcomes between immigrant and non-immigrant children such as in Germany[28], United Kingdom [29], The Netherlands[30], France[31], Spain [6], others have not used the migration status for comparative analyses [32]. Many birth cohorts include only a small proportion of immigrant children, a scoping review reported an average of 10% (ranging from 0 to 60%) of migrant participation in birth cohorts in Europe [32]. In Portugal for instance, only one birth cohort study, established in 2005 is conducted in the northern region of the country (Porto area) with a focus on the study of foetal and childhood determinants in the development of obesity and eventual metabolic changes. This study also examined the relationship between migration and breastfeeding and adverse pregnancy outcomes [8,33]. Like other European birth cohorts, it includes only a small proportion of immigrant children [8].

The CRIAS cohort is the first longitudinal study in the Metropolitan Area of the capital city Lisbon that specifically focuses on gaining a better understanding of the health and development trajectories of immigrant and non-immigrant children, given their respective socioeconomic and cultural contexts. The aim of the CRIAS cohort is to explore whether children exposed to a migratory process, present with different physical health outcomes, cognitive, socioemotional and behavioural challenges and with different health care utilization patterns, over time, when compared to children born in Portugal and raised by parents also born in Portugal. The development of this cohort arises from a strategic and unique partnership between the University, the National Health Service and AJPAS (Associação de Intervenção Comunitária, Desenvolvimento Social e de Saúde) - a local NGO focussing on the needs of immigrant populations. This paper describes the characteristics of the cohort, the baseline cross-sectional study's and the 1st follow-up main findings.

COHORT DESCRIPTION

Setting

The CRIAS study is conducted in the Amadora municipality, in the Metropolitan Area of Lisbon, Portugal. With 171,500 inhabitants in 2021 [34] it is the most densely populated municipality in the country. With a history of immigrants settlement , 13% of its population had a foreign nationality in 2020, making Amadora the second municipality in Portugal, with the highest density of foreign residents – 977/km^2 [1,35]. It is currently served by 10

For peer review only - http://bmjopen.bmj.com/site/about/guidelines.xhtml

Primary Health Care Centres (9 up to December 2020), from now on referred as health centres, and 1 referral hospital – Hospital Fernando Fonseca (HFF). The National Health Service in Portugal (SNS), based on the Beveridge model, is universal and free for children up to the age of 18. Hence, healthcare arrangements in the SNS are the same for all children regardless of their migration status. They include preventive measures such as vaccination and child health monitoring assessments carried out in health centres , as well as specialist and hospital care.

Recruitment and participants

Recruitment was scheduled to take place in the 9 health centres between June 2019-June 2020. However, due to the COVID-19 pandemic, it was discontinued in March 2020. Children were recruited at age 4 to 5 in order for the study to have as many children as possible born outside of Portugal and to be able to identify interventions in all children before school age (6 years). To be included in the study, children had to be born in 2015 and to have records of attending the health centre in the previous 2 years. There were 1009 children with these eligibility criteria in 2019. Based on a previous study [36], we assumed that around 30% of users were immigrant children, i.e.302. In order to maximize comparisons over time between immigrant and non-immigrant children we sought to have the same number of each, resulting in a total of 604 children eligible to participate, together with parents/caregivers.

Families were enrolled while in attendance at the health centre. Recruitment weeks were randomly distributed among the 9 health centres and the number of children recruited was proportional to the number registered in each centre. During recruitment, 499 parents/caregivers were approached; participation rate was 84%. From the 420 children enrolled, 217 were immigrant (51,6%) and 203 non-immigrant, 6 children were twins (4 immigrant, 2 non-immigrant).

At age 5/6 follow-up, children's health centre records showed that 7 children had moved to another municipality. Figure 1 illustrates cohort participation.

Starting in January 2022, families are being contacted by phone to arrange further follow-up assessments

Figure 1. Flow diagram of CRIAS cohort participants.

For peer review only

In order to facilitate enrolment and minimize losses to follow up several strategies were implemented:

- A pilot study (n=33) was conducted to verify acceptability of the questionnaires in terms of content and time by the families
- Active engagement with health professionals. An official presentation of the study to all staff took place in a public venue, followed by further kick-off meetings in each health centre where an interlocutor was nominated to interact with the study team.
- An international team of 6 researchers from 5 different Portuguese speaking countries, and proficient in 6 different languages, was trained to carry out recruitment and conduct initial interviews.
- All participants received details on the study objectives and direct contacts of the research principal investigator. Confidentiality issues and other questions raised were addressed in a culturally sensitive manner by the researchers. Interviews were conducted in total privacy in specially allocated rooms.
- Communication with parents is kept by phone each year and the contacts database is updated regularly with information from the health centres. Feedback (post/email) on screening outcomes is provided and when needed direction to further assessments is given. Face-to-face contact is preferred whenever possible.
- A local NGO -AJPAS working with supporting immigrant communities is involved to facilitate participation

Data collection

The first wave of data collection at age 4/5 was carried out in health centres between June 2019 and March 2020 by a team of 6 researchers using structured questionnaires. All interviewers received the same detailed information and training on the interview process.

Face-to-face interviews were held with parents/caregivers and as a first step we collected family's socioeconomic and demographic characteristics migration history and child health information. The interviews were conducted mostly in Portuguese with other languages used when needed e.g., Creole, English or Asian languages. This was followed by a self-administered screening questionnaire – the Strengths and Difficulties Questionnaire (SDQ), available in validated translations and administered in the preferred language of the participant.

By June 2020, 85% of all COVID-19 infections in Portugal were concentrated in the

For peer review only - http://bmjopen.bmj.com/site/about/guidelines.xhtml

Metropolitan Area of Lisbon, Amadora being one of the most affected municipalities [37]. Lockdown was declared in March 2020. To explore the socioeconomic dynamics of the cohort families during the Covid 19 pandemic, an intermediate data collection was undertaken in July 2020. Phone interviews were conducted applying a semi-structured questionnaire exploring eventual changes in employment and household income, material deprivation and difficulties related to health care access .

The restriction measures adopted during the first waves of the COVID-19 pandemic have limited access to health centres and delayed collection of follow-up data. Nevertheless, baseline (children aged 4/5) and first follow-up (children aged 5/6) clinical data from electronic records for primary care and hospital emergency department visits were collected from November 2019 to October 2021.

Instruments and variables

A schematic representation of instruments and variables used on data collection is shown on figure 2. Parents/caregivers were interviewed using a pilot-tested structured questionnaire to collect sociodemographic information on parents and children; child health history and environmental factors.

The SDQ is a brief questionnaire to assess child emotional and behavioural difficulties, self-administered to one parent or main caregiver, in the parents version for 4-17 years old, translated to Portuguese by Fleitlich and Loureiro [38] as well as in validated translations in other languages. It has been widely used and validated in research, including in multi-ethnic populations of children [39,40] and in several countries including Portugal [41]. Consists of 5 subscales, 4 measuring difficulties which can be grouped into in 2 broad categories of behaviours: externalizing (conduct problems + hyperactivity) and internalizing (emotional + peer problems) behaviours. One subscale measures a strength – prosocial behaviour. A total score of difficulties can also be calculated to classify results as normal, borderline or at risk. Physical health and development information for the ages of 4 and 5 years old was retrieved by two medical doctors who are part of the team from the medical records available on the SCLINICO® primary health care information system. Measurements were performed during the child health monitoring assessments by health professionals in line with the National Child and Youth Health Programme guidelines [42].

Psychomotor development was evaluated by the modified Mary Sheridan screening test, during the health monitoring assessments for ages 4 and 5 [42]. This screening scale is used

For peer review only - http://bmjopen.bmj.com/site/about/guidelines.xhtml

as a reference standard for several skills, distributed in four domains: 1. posture and global motor skills; 2. vision and fine motor skills; 3. hearing and language; 4. behaviour and social adaptation. Outcomes of the test were categorized in “monitoring not required ” if all items were fulfilled and in “monitoring required “if one or more items was not achieved and required a review, usually carried out in 6 months’ time. Additional variables from electronic medical records were related to access and utilization of health care services; data on utilization of the emergency department was provided by the hospital.

Figure 2. Representation of data collection and main variables of the CRIAS cohort study

Data linkage

Electronic medical records are managed in primary health care and in the hospital in two different information systems, SCLINICO and SORIAN respectively. We link 3 different datasets: data collected through interviews; primary health care data and hospital data. The information is obtained from the electronic medical records with the SNS number which is then returned to the project coordinator who matches this number with the identification code (ID) of the child. The key which assigns the SNS user number to the name of the child and to the ID code is password protected and kept by the coordinator of the study. The integrated cohort database available for analysis only includes the ID code.

Patient and public involvement

The NGO AJPAS, founded by immigrants and located in Amadora Municipality, and the members of the regional health authorities have been involved in the design, governance and general oversight of all phases of the research to date. Study participants have been encouraged to communicate to the research team by phone and email. Reports and presentations are frequently shared with key stakeholder groups. Members of the NGO AJPAS have been trained to also participate as interviewers in the survey on the socioeconomic impact of the COVID-19 pandemic providing economic, social and legal support whenever requested by the study participants.

FINDINGS TO DATE

Baseline characteristics of children and parents/caregivers

A large majority of the 417 parent/caregivers interviewed were women (88%), nearly all were the mothers. The main countries of origin of immigrant parents/caregivers were Cape Verde (n=60), Angola (n=28), Brazil (n=28), and Guinea-Bissau (n=22). The main reasons for

For peer review only - http://bmjopen.bmj.com/site/about/guidelines.xhtml

immigration given by the mother were family reunification (28.9%), obtaining a better education (27.6%), economic reasons (22.4%), with 3.3% having moved because of war; the median length of stay in years in Portugal was 9 years (min.0.1-max.37). Information on the main sociodemographic characteristics of the families is found in table 1.

Table 1. Baseline characteristics of the parents/caregivers of children in the CRIAS cohort

**Parents/caregivers n=417**	**Immigrant children n (%)**	**Non-immigrant children n (%)**	**Total n (%)**	**p value**
**Sex**				
Female	187 (86.6)	179 (89.1)	366 (87.8)	0.440*
**Age n=417**				0.213**
Median (min-max; IQR)	34 (20-75;10)	35 (18-68;10)	35(18-75;10)	
**Relationship with child n=417**				0.262*
Mother	182 (84.3)	177 (88.1)	359 (86.1)	
Others	34 (15.7)	24 (11.9)	58 (13.9)	
**Educational level [a] n=416**				0.115*
Lower education	40 (18.6)	27 (13.4)	67 (16.1)	
9 years completed	41 (19.1)	45 (22.4)	86 (20.7)	
Secondary education	91 (42.3)	73 (36.3)	164 (39.4)	
University degree	43 (20.0)	56 (27.9)	99 (23.8)	
**Occupation [b] n= 414**				<0.001*
High skilled	34 (16.0)	69 (34.3)	103 (24.9)	
Medium skilled	99 (46.5)	102 (50.7)	201 (48.6)	
Low skilled	75 (35.2)	20 (10.0)	95 (22.9)	
Non- defined	5 (2.3)	10 (5.0)	15 (3.6)	
**Employment status n=417**				0.009*
Employed with a contract	135 (62.5)	157 (78.1)	292 (70.0)	
Employed without a contract	20 (9.3)	5 (2.5)	25 (6.0)	
Unemployed with benefits	13 (6.0)	10 (5.0)	23 (5.5)	
Unemployed without benefits	18 (8.3)	11(5.5)	29 (7.0)	
Self-employed	16 (7.4)	9 (4.5)	25 (6.0)	
Other [c]	14 (6.5)	9 (4.5)	23 (5.5)	
**Household monthly income n=395**				<0.001*
<500 €	39 (19.1)	12 (6.3)	51 (12.9)	
>500—750	66 (32.4)	44 (23.0)	110 (27.8)	
>750—1000€	38 (18.6)	34 (17.8)	72 (18.2)	

For peer review only - http://bmjopen.bmj.com/site/about/guidelines.xhtml

>1000—1500€	36 (17.6)	43 (22.5)	79 (20.0)
>1500—2000€	16 (7.8)	24 (12.6)	40 (10.1)
>2000€	9 (4.4)	34 (17.8)	43 (10.9)

Significance level 5%. *Pearson- Chi square statistical test ** Mann-Whitney U statistical test

[a] based on the International Standard Classification of Education[43]; [b] classified as per the Portuguese Classification of Professions and summarized in 4 skill levels according to the International Standard Classification of Occupations [44]; [c] students, stay-at-home parents, retired

Information collected on 217 (51,6%) immigrant children in the cohort, showed that 41 children were born in a non-EU country. They originated mainly from the Community of Portuguese Speaking Countries: Brazil (13), Angola (8), Guinea-Bissau (6) and Cape Verde (4). The median length of stay in Portugal of these 1st generation immigrant children was 18 months (1 min.- 48 max.). Children from countries such as India, Nepal or Eritrea are also present in the study. Portuguese is spoken in 268 households (64%) while 17 other languages, ranging from Nepalese to Mandarin and Tigrinya are spoken in the remaining households. After the Portuguese, the most common language spoken is a combination of Creole and Portuguese (20%), spoken not only by immigrant families but also in 6.4% of households of non-immigrant children, suggesting a possible migration background of the grandparents. Table 2 shows other relevant characteristics of participating children.

Table 2 . Main characteristics of children in the CRIAS cohort at baseline age 4/5

**Characteristics of the children**	**Immigrant**	**Non-immigrant**	**Total**	**p value**
	**n (%)**	**n (%)**	**n (%)**	
	217 (51.7)	203 (48.3)	420 (100)	
**Sex n=420**				0.689*
Female	109 (50.2)	98 (48.3)	207 (49.3)	
**Gestational age n=413**				0.463*
<37weeks – Preterm	15 (7.0)	18 (9.0)	33 (8.0)	
>37 weeks	198 (93)	182 (91)	380 (92)	
**Birthweight n=385**				0.531*
<2500g– Low Birth Weight	19 (9.9)	16 (8.1)	35 (9.0)	
>2500g	172 (90.1)	181 (91.9)	353 (91.0)	
**Breastfeeding n=419**	203 (93.1)	179 (89.1)	382 (91.2)	0.152*
Total duration breast feeding n=375 Median months (min-max; IQR)	12 (0-53;18)	6 (0-48;14)	10 (0-53;15)	<0.001**

For peer review only - http://bmjopen.bmj.com/site/about/guidelines.xhtml

**Family structure n=419**				0.084*
Both parents	99 (45.8)	117 (57.6)	216 (51.6)	
Both parents and others	30 (13.9)	27 (13.3)	57 (13.6)	
Single-parent families	42 (19.4)	28 (13.8)	70 (16.7)	
One parent and others/others	45 (20.9)	31 (15.3)	76 (18.1)	
**Large households (>=5 people) n=420**	79 (36.2)	57 (28.2)	136 (32.4)	0.079*
**Ratio people in household/ number bedrooms n=420** Mean (95% CI)	2.00 (1.89 to 2.11)	1.73 (1.65 to 1.81)	1.87 (1.80 to 1.94)	<0.001***
**Childcare arrangements n=417**				0.329*
State pre-school	83 (38.4)	81 (39.9)	164 (39.1)	
Private pre-school	94 (43.5)	98 (48.3)	192(45.8)	
Stays home w/mother	15 (6.9)	8 (3.9)	23 (5.5)	
Other	24 (11.1)	16 (7.9)	40 (9.5)	
**Assigned family doctor n=420**	161(73.9)	179 (88.6)	340 (81.0)	<0.001*
**Private Health Insurance n=417**	63 (29.3)	104 (51.5)	104 (51.5)	

Significance level 5%. *Pearson- Chi square statistical test ** Mann-Whitney U statistical test *** t-test

Findings on key outcomes and other variables at ages 4/5 and 5/6

At baseline (age 4/5) the perceived health of the child was considered to be very good or good by 80% of the parents. The median number of parent reporting episodes of illness in the last 3 months was one and did not differ between immigrant and non-immigrant children. Most frequently reported complaints, for immigrant and non-immigrant children respectively, were related to cough and other symptoms of the respiratory tract 49.5% vs. 66.5%, fever 18.8% vs. 17.3 %, skin problems 6.4% vs. 4% and digestive complains 7.9% vs. 6.5%. Vaccination rates were above 90% for all children. In the Modified Mary Sheridan test to evaluate psychomotor development, more immigrant children were found to require monitoring of one or more items (38%vs. 28%). In both groups, 25% of children required monitoring in items on the vision and fine motor skills domain. The above information is shown on supplementary table 1. The findings on emotional and behavioural difficulties suggest that a low family income (aOR 4.5; 95%CI: 1.43-13.95), low parental education level (aOR 2.5; 95%CI: 1.11- 5.16) and being a 1st generation immigrant child (aOR 2.2; 95%CI: 1.06-4.76) may increase significantly the odds of developing emotional and behavioural difficulties; these results are shown on supplementary table 1b.

For peer review only

The main variables collected on health services utilization are summarized in table 3. Over a quarter (26%) of immigrant children did not have a regular allocated family doctor and 36% of 1st generation immigrants did not receive the health monitoring assessments for age 4 Non-immigrant children used less primary care (73.8% vs. 78%), the hospital emergency department was more used by immigrant children (53.2% vs. 40.6%).

Other findings at age 4/5 (supplementary table 2.) included dental caries observed in 23% of the children with a similar number having vision acuity or eyes alignment difficulties, with no differences among groups. The recommended intake of fruits and vegetables is not achieved by most children, particularly immigrant children. Overweight was found in 25% of the children (22% in immigrant vs. 28% in non-immigrant children), 6% of children were obese and from a total of 8% underweight children, most were immigrant.

The main results from the additional module (July 2020) on the potential socioeconomic effects of COVID-19 pandemic on families participating in the CRIAS-cohort study are shown in supplementary table 3. Immigrant parents were more likely to be unemployed due to the COVID-19 pandemic (aOR 3.54, 95% CI 1.72-7.30) and more likely to have their household income decreased (aOR 3.21, 95% CI 1.80-5.75).

At age 5 follow-up, during the 1st year of the COVID-19 pandemic, about 2/3 of all children did not receive routine assessments for age 5, mostly due to limited access to the health centres as a result of the pandemic restrictions. Immigrant children continued to require greater attention on their psychomotor development with 33.9% versus 21.6% non-immigrant children having test items with monitoring required monitoring.

Emergency department use dropped significantly (28.9% for immigrant children vs. 26.7 %).

Table 3 compares health care utilization at ages 4 and 5.

Table 3. Utilization of healthcare services by children in the CRIAS cohort at age 4 and 5

	**1st wave of data collection (age 4) n=420**				**2nd wave of data collection (age 5) n=420**			
	**Immigrant** n (%)	**Non-immigrant** n (%)	**Total** n (%)	**p value**	**Immigrant** n (%)	**Non-immigrant** n (%)	**Total** n (%)	**p value**
**Primary care**	**At least one consultation in 2019**			0.312*	**At least one consultation in 2020**			0.018*
	170 (78.0)	149 (73.8)	319 (76.0)		153 (70.2)	162 (80.2)	315 (75.0)	
	**Most frequent diagnosis in 2019[1]**				**Most frequent diagnosis in 2020[1]**			

For peer review only - http://bmjopen.bmj.com/site/about/guidelines.xhtml

**Respiratory infections**	52 (24.0)	58 (28.6)	110 (26.2)	0.283*	20 (9.2)	33 (16.3)	53 (12.6)	0.027*
**Skin**								
Parasitic and fungal infections	8 (3.7)	3 (1.5)	11(2.6)	0.157**	7 (3.2)	3 (1.5)	10 (2.4)	0.341**
Atopic dermatitis	31 (14.3)	14 (6.9)	45 (10.7)	0.014*	17 (7.8)	12 (5.9)	29 (6.9)	0.453
**Digestive**								
Gastro-enteritis	11 (5.1)	11 (5.4)	22(5.2)	0.872*	5 (2.3)	5 (2.5)	10 (2.4)	0.903*
Others	23 (10.6)	13 (6.4)	36 (8.6)	0.125*	16 (7.3)	16 (5.9)	32 (7.6)	0.822*
	**Health monitoring assessment at age 4**				**Health monitoring assessment at age 5**			
	161 [c] (74.2)	160 (78.8)	321 (76.4)	0.265*	72 [d] (33.2)	64 (31.5)	136 (32.4)	0.718*
**Hospital**	**At least one Emergency Department visit in Year 2019**				**At least one Emergency Department visit in Year 2020**			
	116 (53.5)	82 (40.4)	198 (47.1)	0.007*	63 (29.0)	54 (26.6)	117 (27.9)	0.579*
	14.7 (32)	6.4 (13)	10.7 (45)	0.006*	1.4 (3)	2.0 (4)	1.7 (7)	0.716**

[1] based on the International Classification of Primary Care (ICPC-2) clinical activity for the health centres
[a] number of consultations in 2019,n=735; [b] number consultations 2020,n=606

Significance level 5%. *Pearson- Chi square statistical test ; ** Fisher's exact test

[c]36.6% 1st generation immigrant children did not received the routine health assessment for 4 year olds

[d]75.6% 1st generation immigrant children did not received the routine health assessment for 5 year olds

The findings on emotional and behavioural difficulties have been published [45]; general findings from the 1st wave of data have been published as an abstract in the European Journal of Public Health [46]. Results from the survey on the socioeconomic impact of COVID-19 on immigrant and non-immigrants families were awarded with the Human Rights Gold Medal Prize given in 2020 by the National Assembly of the Republic of Portugal; main findings have been submitted to a scientific journal and are under review.

FUTURE PLANS

We are preparing to resume face to face contacts with families and are already contacting the participants by phone. Selected socioeconomic information will be updated. Considering the recent rise in mental health difficulties in children, related with the COVID-19 pandemic, the follow up SDQ assessment might reveal new developments. An additional

For peer review only

For peer review only - http://bmjopen.bmj.com/site/about/guidelines.xhtml

module to Study Asthma and Allergies in Childhood will be implemented at age 6/7, using ISAAC Methodology [47]. Information on the experience of accessing and using health services by the immigrant children in the study and their families will be complemented by a qualitative study. Further follow-ups will be carried out at the key ages of 8, 10, 12/13 and 15/18 of the National Child and Youth Health Programme [42] if ongoing financing.

The conduct of this study and its societal implications led us to extend the study to another 4 Municipalities in the Lisbon Region with the collection of data on further 900 children (450 immigrant) through a sequential-cohort design, likely to include more immigrants from non lusophone countries. Funded by the National Science Foundation, in partnership with 2 local NGOs (AJPAS and Doctors of the World) and 15 health centres, the extension of the cohort study will start in February 2022 .

We will continue to disseminate our results in conferences, scientific papers and meetings with local NOGs and policy makers at the Regional level. A book is in preparation for the Migrations Observatory in Portugal.

Although Portugal provides free healthcare for all children including undocumented migrants and repeatedly scores high in migrant integration policies, MIPEX 2020 [48] the Migration Policies Index gives a less favourable score for healthcare. Therefore, we will continue to work to translate our findings into policies and services change to improve access, quality of healthcare provision and contribute to better lives of all children.

STRENGTHS AND LIMITATIONS OF THIS STUDY

Our study presents several strengths. First and foremost, CRIAS is the first cohort study in Metropolitan Area of the capital city Lisbon, Portugal, created to specifically address the role of immigration as a physical and emotional health determinant, by comparing outcomes in immigrant and non-immigrant children over time. In contrast to many cohort studies initiated in the EU which report results regarding immigration’s potential impacts while including on average 10% of children with a migration background, this cohort study includes about 50%. This proportion of immigrant children in the sample increases the power of comparisons between immigrant and non-immigrant children over time compared to other studies. Due to the colonial past of Portugal, these immigrant populations are mainly from Brazil and Portuguese speaking African countries who might have diverse

For peer review only

migration experiences, biological and cultural factors and health utilization behaviours than the populations included in other cohort studies in Europe.

The partnership with a local NGO is critical in the recruitment and follow-up phases and in providing direct support to immigrant families and their children during COVID-19 times. Timely presentation of the results to primary health care professionals potentiates the identification of early interventions. The start of the project just before the COVID-19 pandemic made it possible to follow and, whenever possible, support (via the NGO partnership) more vulnerable families during the pandemic crisis.

One limitation may appear during the next follow-up steps because of the early interruption of recruitment of eligible immigrant children (expected n=302) due to lockdown and social distancing restrictions required by the COVID-19 pandemic, resulting in a smaller sample size. Difficulties on re-establishing face to face contact with families and on providing the child health monitoring assessments by health professionals, often under constrained time and resources in the pandemic context, have delayed follow-up data collection. Another limitation is the absence of information on children who don't attend public primary health care centres which limits the representativeness of the study to those who attend public primary care.

COLLABORATION

Initial data analysis and publications will be generated by investigators on CRIAS cohort research team . Study data is not currently freely available .However, deidentified data are available upon reasonable request from the Coordinator of the study MROM - ORCID ID: 0000-0002-7941-0285. The research team welcomes collaboration with other researchers.

Authors affiliation:

[1] Global Health and Tropical Medicine, Institute of Hygiene and Tropical Medicine, NOVA University, Lisbon, Portugal

[2] Interdisciplinary Centre of Social Sciences (CICS.NOVA), Faculty of Social Sciences and Humanities (NOVA FCSH), Lisbon, Portugal

[3] Amadora Primary Care Health Centres Group, Regional Health Administration of Lisbon and Tagus Valley, Amadora, Portugal

[4] Department of Paediatrics, Hospital Prof. Dr. Fernando Fonseca, Amadora , Portugal

For peer review only

[5] Public Health Department, Regional Health Administration of Lisbon and Tagus Valley, Lisbon, Portugal

[6] AJPAS, NGO, Associação de Intervenção Comunitária, Desenvolvimento Social e de Saúde, Amadora, Portugal

Acknowledgments: We would like to thank all participant families and health centre's staff. The authors acknowledge all the support received from the Executive Direction and Clinical Advisory Board of Amadora group of health centers and Hospital Prof. Doutor Fernando Fonseca and the NGO AJPAS.

Author Contributions: Conceptualization, MROM, ZM, TM, ALT, DV, ACS, IF, AA; methodology, MROM, ZM, TM, IF; validation, MROM, ZM, RA, MP, HL; formal analysis, MROM, ZM, RA; data analysis, MROM, ZM, RA; writing—original draft preparation, ZM, MROM; writing—review and editing, MROM, ZM, TM, IF, AA, DV, AA, ACS; data collection, ZM, RA, MROM, MP, HL; supervision, MROM; project administration, MROM; funding acquisition, MROM. All authors have read and agreed to the published version of the manuscript.

Ethics approval: Health Ethic Committee of the Regional Health Administration of Lisbon and Tagus Valley, Portugal (001/CES/INV/2019), including an additional approval for the COVID-19 intermediate study (9-2020/CES/2020). A written information and consent form to participate in the study was signed by the parents, which included permission to assess data from the child's health centre and hospital medical records

Funding: This research was financed by the Asylum, Integration and Migration Fund (PT/2018/FAMI/350), by the National Science Foundation (RESEARCH4COVID-19-065) and by the Multiannual Financial Framework 2014/20, Global Health and Tropical Medicine (GHTM), Institute of Hygiene and Tropical Medicine (IHMT), NOVA University of Lisbon, Portugal, reference: UID/04413/2020. The extension of the cohort study is financed by the National Science Foundation (PTDC/SAU-SER/4664/2020).

Patient consent for publication: Not required

Conflicts of Interest: The authors declare no conflict of interest. The funders had no role in the design of the study; in the collection, analyses, or interpretation of data; in the writing of the manuscript, or in the decision to publish the results.

For peer review only - http://bmjopen.bmj.com/site/about/guidelines.xhtml

Data availability statement. Deidentified participant data are available upon reasonable request from the Coordinator of the study MROM - ORCID ID: 0000-0002-7941-0285

REFERENCES

1. SEF/GEPF. Relatório de Imigração, Fronteiras e Asilo 2020 [Internet]. Oeiras, Portugal: Serviço de Estrangeiros e Fronteiras; 2021 Jun. Available from: https://sefstat.sef.pt/Docs/Rifa2020.pdf

2. Alto Comissariado para as Migrações. Plano estratégico para as migrações (2015-2020). [Internet]. Alto Comissariado para as Migrações , Lisboa; Available from: https://www.acm.gov.pt/documents/10181/222357/PEM_net.pdf/3a515909-7e66-41e8-8179-e3aa5e0c7195

3. Abubakar I, Aldridge RW, Devakumar D, Orcutt M, Burns R, Barreto ML, et al. The UCL–Lancet Commission on Migration and Health: the health of a world on the move. *The Lancet* [Internet]. 2018 Dec [cited 2019 Mar 14];392(10164):2606–54. Available from: https://linkinghub.elsevier.com/retrieve/pii/S0140673618321147

4. WHO European Region. Report on the health of refugees and migrants in the WHO European Region: no public health without refugee and migrant health (2018) [Internet]. WHO; Available from: https://apps.who.int/iris/bitstream/handle/10665/311347/9789289053846-eng.pdf?sequence=1&isAllowed=y

5. Gualdi-Russo E, Toselli S, Masotti S, et al. Health, growth and psychosocial adaptation of immigrant children. *European Journal of Public Health* [Internet]. 2014 Aug 1 [cited 2021 Oct 21];24(suppl_1):16–25. Available from: https://doi.org/10.1093/eurpub/cku107

6. Ferrer L, Gaillardin F, Cayuela A, et al. Children health determinants in a multi-site cohort of immigrant families in Spain (PELFI Study). *European Journal of Public Health* [Internet]. 2016 Nov [cited 2021 Nov 13];26(suppl_1). Available from: https://academic.oup.com/eurpub/article-lookup/doi/10.1093/eurpub/ckw175.114

For peer review only

7. Dias S. Atitudes e representações face à saúde, doença e acesso aos cuidados de saúde nas populações imigrantes. Lisboa: Alto Comissariado para as Migrações; 2018.

8. Kana MA, Rodrigues C, Fonseca MJ, et al. Effect of maternal country of birth on breastfeeding practices: results from Portuguese GXXI birth cohort. *International Breastfeeding Journal* [Internet]. 2018 Apr 10 [cited 2022 Jul 3];13(1):15. Available from: https://doi.org/10.1186/s13006-018-0157-x

9. Relatórios Estatísticos Anuais - om [Internet]. [cited 2022 Jul 13]. Available from: https://www.om.acm.gov.pt/publicacoes-om/colecao-imigracao-em-numeros/relatorios-anuais

10. Carrasco-Sanz A, Leiva-Gea I, Martin-Alvarez L et al. Migrant children's health problems, care needs, and inequalities: European primary care paediatricians' perspective. *Child: Care, Health and Development* [Internet]. 2018 [cited 2020 Apr 5];44(2):183–7. Available from: https://onlinelibrary.wiley.com/doi/abs/10.1111/cch.12538

11. Malick G, Fabienne J, Noémie W, Mario G. Primary care for the migrant population in Switzerland: a paediatric focus. 27(2016):7.

12. Hjern A. Health of refugee and migrant children : Technical guidance [Internet]. 2018 [cited 2020 Sep 8]. Available from: http://urn.kb.se/resolve?urn=urn:nbn:se:su:diva-164328

13. Jaeger FN, Hossain M, Kiss L, Zimmerman C. The health of migrant children in Switzerland. *Int J Public Health*. 2012 Aug;57(4):659–71.

14. Markkula N, Cabieses B, Lehti V et al. Use of health services among international migrant children – a systematic review. *Global Health* [Internet]. 2018 Dec [cited 2020 Mar 9];14(1):52. Available from: https://globalizationandhealth.biomedcentral.com/articles/10.1186/s12992-018-0370-9

15. Pavlopoulou ID, Michail KA, Samoli E et al. Immunization coverage and predictive factors for complete and age-appropriate vaccination among preschoolers in Athens,

For peer review only

Greece: a cross- sectional study. *BMC Public Health* [Internet]. 2013 Dec [cited 2021 Oct 22];13(1):908. Available from: http://bmcpublichealth.biomedcentral.com/articles/10.1186/1471-2458-13-908

16. Poethko-Müller C, Ellert U, Kuhnert R,et al. Vaccination coverage against measles in German-born and foreign-born children and identification of unvaccinated subgroups in Germany. *Vaccine* [Internet]. 2009 Apr 28 [cited 2021 Oct 22];27(19):2563–9. Available from: https://www.sciencedirect.com/science/article/pii/S0264410X09002072

17. Riise ØR, Laake I, Bergsaker MAR et al. Monitoring of timely and delayed vaccinations: a nation-wide registry-based study of Norwegian children aged < 2 years. *BMC Pediatrics* [Internet]. 2015 Nov 13 [cited 2021 Oct 22];15(1):180. Available from: https://doi.org/10.1186/s12887-015-0487-4

18. Leth I, Niclasen J, Ryding E et al. Psychological difficulties among children and adolescents with ethnic Danish, immigrant or refugee background. *Scandinavian Journal of Child and Adolescent Psychiatry and Psychology* [Internet]. 2014 Mar 25 [cited 2020 May 9];2(1):29–36. Available from: https://tidsskrift.dk/sjcapp/article/view/15814

19. Belhadj Kouider E, Koglin U, Petermann F. Emotional and behavioral problems in migrant children and adolescents in Europe: a systematic review. *Eur Child Adolesc Psychiatry* [Internet]. 2014 Jun 1 [cited 2020 Dec 4];23(6):373–91. Available from: https://doi.org/10.1007/s00787-013-0485-8

20. Kouider EB, Lorenz AL, Dupont M, et al.Internalizing disorders in migrant and non-migrant children and adolescents: analyses of a German health care population. *Journal of Public Health* [Internet]. 2015 [cited 2022 Jul 12];6(23):349–61. Available from: https://www.infona.pl//resource/bwmeta1.element.springer-doi-10_1007-S10389-015-0688-1

21. Vollebergh WAM, ten Have M, Dekovic M, et al. Mental health in immigrant children in the Netherlands. Soc Psychiatry Epidemiol. 2005 Jun;40(6):489–96.

For peer review only - http://bmjopen.bmj.com/site/about/guidelines.xhtml

22. World Health Organization Early Child Development Knowledge Network (WHO ECDKN). Early Child Development: A Powerful Equalizer. Final Report of the Early Childhood Development Knowledge Network of the Commission on Social Determinants of Health. Geneva: World Health Organization, 2007.

23. Pillas D, Marmot M, Naicker K,et al. Social inequalities in early childhood health and development: a European-wide systematic review. *Pediatr Res.* 2014 Nov;76(5):418–24.

24. Marmot M. Health equity in England: the Marmot review 10 years on. *BMJ* [Internet]. 2020 Feb 24 [cited 2020 Nov 9];m693. Available from: https://www.bmj.com/lookup/doi/10.1136/bmj.m693

25. Jaddoe VWV, Felix JF, Andersen AMN, et al. The LifeCycle Project-EU Child Cohort Network: a federated analysis infrastructure and harmonized data of more than 250,000 children and parents. *Eur J Epidemiol* [Internet]. 2020 [cited 2022 Jul 4];35(7):709–24. Available from: https://www.ncbi.nlm.nih.gov/pmc/articles/PMC7387322/

26. Balbus JM, Barouki R, Birnbaum LS, et al. Early-life prevention of non-communicable diseases. *The Lancet* [Internet]. 2013 Jan 5 [cited 2022 Jul 4];381(9860):3–4. Available from: https://www.thelancet.com/journals/lancet/article/PIIS0140-6736(12)61609-2/fulltext

27. Wachs TD, Georgieff M, Cusick S, et al.Issues in the timing of integrated early interventions: contributions from nutrition, neuroscience and psychological research. *Ann N Y Acad Sci* [Internet]. 2014 Jan [cited 2022 Jun 29];1308:89–106. Available from: https://www.ncbi.nlm.nih.gov/pmc/articles/PMC4075015/

28. Spallek J, Grosser A, Höller-Holtrichter C, et al. Early childhood health in Bielefeld, Germany (BaBi study): study protocol of a social-epidemiological birth cohort. *BMJ Open* [Internet]. 2017 Aug 1 [cited 2022 Jul 4];7(8):e018398. Available from: https://bmjopen.bmj.com/content/7/8/e018398

For peer review only

29. Bird PK, McEachan RRC, Mon-Williams M, et al. Growing up in Bradford: protocol for the age 7–11 follow up of the Born in Bradford birth cohort. *BMC Public Health* [Internet]. 2019 Jul 12 [cited 2022 Jul 4];19(1):939. Available from: https://doi.org/10.1186/s12889-019-7222-2

30. Jaddoe VWV, Mackenbach JP, Moll HA, et al. The Generation R Study: Design and cohort profile. *Eur J Epidemiol* [Internet]. 2006 Jul 7 [cited 2022 Jul 4];21(6):475. Available from: https://doi.org/10.1007/s10654-006-9022-0

31. Castillo Rico B, Leturcq M, Panico L. La pauvreté des enfants à la naissance en France. Résultats de l'enquête Elfe. *Revue des politiques sociales et familiales* [Internet]. 2019 [cited 2022 Jul 4];131(1):35–49. Available from: https://www.persee.fr/doc/caf_2431-4501_2019_num_131_1_3342

32. Grosser A, Razum O, Vrijkotte TGM, et al. Inclusion of migrants and ethnic minorities in European birth cohort studies—a scoping review. *Eur J Public Health* [Internet]. 2016 Dec [cited 2020 Dec 4];26(6):984–91. Available from: https://academic.oup.com/eurpub/article-lookup/doi/10.1093/eurpub/ckw068

33. Kana MA, Correia S, Barros H. Adverse Pregnancy Outcomes: A Comparison of Risk Factors and Prevalence in Native and Migrant Mothers of Portuguese Generation XXI Birth Cohort. *J Immigr Minor Health.* 2019 Apr;21(2):307–14.

34. Statistics Portugal. INE - Plataforma de divulgação dos Censos 2021 – Resultados Provisórios [Internet]. [cited 2022 Jan 6]. Available from: https://ine.pt/scripts/db_censos_2021.html

35. PORDATA - População estrangeira com estatuto legal de residente: total e por algumas nacionalidades [Internet]. [cited 2020 Sep 8]. Available from: https://www.pordata.pt/Municipios/Popula%C3%A7%C3%A3o+estrangeira+com+estatuto+legal+de+residente+total+e+por+algumas+nacionalidades-101

36. Jonas JA. The impact of economic crisis on children's health: analysis of the perceptions of migrant and native families'users of primary health care in Amadora [Internet]

For peer review only

[masterThesis]. Instituto de Higiene e Medicina Tropical; 2018 [cited 2021 Oct 23]. Available from: https://run.unl.pt/handle/10362/52853

37. Direção-Geral da Saúde sobre o Novo Coronavírus COVID-19. Ministra da Saúde anuncia medidas específicas para a região de Lisboa [Internet]. COVID-19. [cited 2021 Nov 13]. Available from: https://covid19.min-saude.pt/ministra-da-saude-anuncia-medidas-especificas-para-a-regiao-de-lisboa/

38. Fleitlich, B., Loureiro, M., Fonseca, A., & Gaspar, F. (2005). Questionário de capacidades e dificuldades (SDQ-Por) [Strengths and Difficulties Questionnaire, Portuguese Version]. Retrieved from www.sdqinfo.org. Questionário de capacidades e dificuldades (SDQ-Por) [Strengths and Difficulties Questionnaire, Portuguese Version]. [Internet]. Available from: www.sdqinfo.org

39. Mieloo CL, Bevaart F, Donker MCH, et al. Validation of the SDQ in a multi-ethnic population of young children. *Eur J Public Health* [Internet]. 2014 Feb 1 [cited 2020 Apr 9];24(1):26–32. Available from: https://academic.oup.com/eurpub/article/24/1/26/493856

40. Runge RA, Soellner R. Measuring children's emotional and behavioural problems: are SDQ parent reports from native and immigrant parents comparable? *Child and Adolescent Psychiatry and Mental Health* [Internet]. 2019 Nov 28 [cited 2020 Sep 3];13(1):46. Available from: https://doi.org/10.1186/s13034-019-0306-z

41. Costa PA, Tasker F, Ramos C, et al. Psychometric properties of the parent's versions of the SDQ and the PANAS-X in a community sample of Portuguese parents. *Clin Child Psychol Psychiatry* [Internet]. 2020 Apr [cited 2020 Apr 9];25(2):520–32. Available from: http://journals.sagepub.com/doi/10.1177/1359104519891759

42. Programa Nacional de Saúde Infantil e Juvenil [Internet]. [cited 2021 Oct 24]. Available from: https://www.dgs.pt/documentos-e-publicacoes/programa-tipo-de-atuacao-em-saude-infantil-e-juvenil.aspx

43. UNESCO Institute for Statistics. International standard classification of education: ISCED 2011 [Internet]. Montreal, Quebec: UNESCO Institute for Statistics; 2012 [cited

For peer review only - http://bmjopen.bmj.com/site/about/guidelines.xhtml

2021 Oct 24]. Available from: http://www.uis.unesco.org/Education/Documents/isced-2011-en.pdf

44. International Labour Office. International Standard Classification of Occupations: ISCO-08. ILO, Geneva; 2012.

45. Muggli Z, Mertens T, -Sá S, et al. Migration as a Determinant in the Development of Children Emotional and Behaviour Problems: A Quantitative Study for Lisbon Region, Portugal. *Int J Environ Res Public Health* [Internet]. 2021 Jan [cited 2021 Oct 24];18(2):375. Available from: https://www.ncbi.nlm.nih.gov/pmc/articles/PMC7825293/

46. Muggli Z, Amado R, Oliveira Martins MR, et al. The health of immigrant children: a cohort study in the Metropolitan area of Lisbon - Portugal. *European Journal of Public Health* [Internet]. 2020 Sep 1 [cited 2022 Jan 9];30(Supplement_5):ckaa166.787. Available from: https://doi.org/10.1093/eurpub/ckaa166.787

47. Ellwood P, ISAAC Steering Committee, ISAAC Phase Three Study Group, ISAAC International Data Centre. ISAAC: International Study of Asthma and Allergies in Childhood : phase three manual. Auckland, N.Z.: ISAAC International Data Centre; 2000.

48. Portugal | MIPEX 2020 [Internet]. www.mipex.eu. [cited 2021 Oct 24]. Available from: https://www.mipex.eu/portugal

For peer review only

For peer review only - http://bmjopen.bmj.com/site/about/guidelines.xhtml

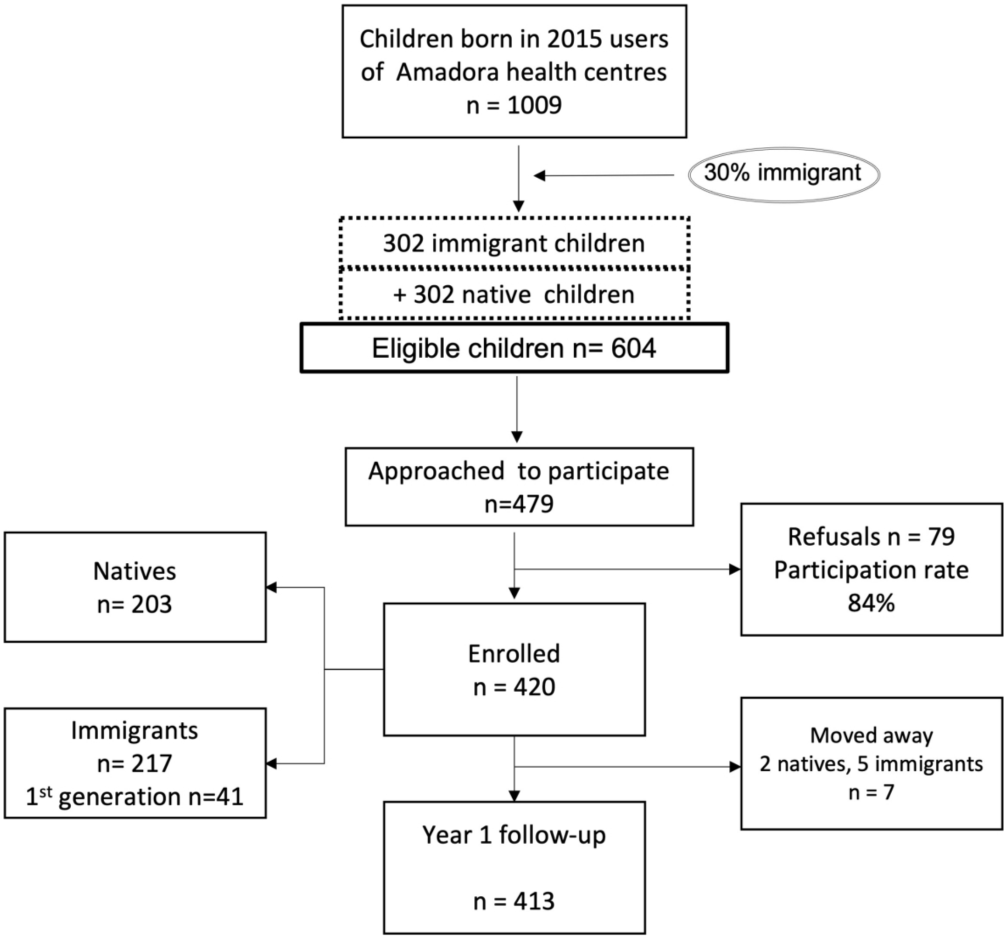

Figure 1. Flow Diagram of CRIAS Cohort Participants

178x166mm (300 x 300 DPI)

Variables		4/5 years old Jun 19-Mar 20	Intermediate data July 2020	5/6 years old	6/7 years old 2022 (ongoing)
**Parents /caregivers**	**Child**				
**Sociodemographic**<br>Sex, age, country of birth<br>Education, occupation, employment, monthly income<br>Private health insurance<br>Migration history; length of stay	**Sociodemographic**<br>Sex<br>Country of birth, length of stay in Portugal				
	**Health history**<br>Birth details and breast feeding duration<br>Dietary intake<br>**Perceived health by parents ***<br>**Illness episodes in last 3 months ***				
	**Environmental factors**<br>Language spoken at home<br>Family composition; household size<br>Ratio persons/number of bedrooms<br>Pre-school/childcare arrangements				
	**Physical health**<br>BMI<br>Dental caries<br>Vision<br>**Vaccination ***				
	**Psychomotor development ***				
	**Emotional and behavioural difficulties***				
	**Access and utilization of healthcare services ***<br>Assigned family doctor<br>Primary care consultations<br>Hospital emergency visits				
**Socioeconomic changes in children´s families due to COVID-19 pandemic**<br>Changes in employment and household income<br>Changes in material deprivation<br>Difficulties related to the lockdown	*Key outcomes				

figure2. Data Collection and variables of the CRIAS cohort Study

249x241mm (300 x 300 DPI)

Supplementary table 1a. Key health oucomes for children at age 4/5 years

	Immigrant	Non-immigrant	Total	*p* value
	n (%)	n (%)	n (%)	
	217 (51.7)	203 (48.3)	420 (100)	
**Perceived health by parents** n=415				0.986*
Very good and good	173 (80.1)	160 (80.4)	333 (80.2)	
Fair	39 (18.1)	35 (17.6)	74 (17.8)	
Bad and very bad	4 (1.9)	4 (2.0)	8 (1.9)	
**Illness episodes in previous 3 months (parent reported)** n=410				0.340**
Median (min-max;IQR)	1 (0-5;1)	1 (0-5;2)	1(0-5;1)	
**Psychomotor development** n=281				
All items achieved	88 (61.5)	99 (71.7)	187 (66.5)	0.077*
To monitor	55 (38.5)	39 (28.3)	94 (33.5)	
**Emotional and behavioural difficulties** n=420				
Median externalizing behaviours (min.-max.;IQR)	7 (0-18; 5)[a]	7.5 (1-20; 5)	7 (0-20; 5)	0.950**
Median internalizing behaviours (min.-max.;IQR)	4 (0-13; 4)	3 (0-15; 3)	4 (0-15;4)	p<0.001**
**Vaccination up to date** n=416				p=0.852*
yes	195 (91.1)	183 (90.6)	378 (90.9)	
no	19 (8.9)	19 (9.4)	38 (9.1)	

Significance level 5%. *Pearson- Chi square statistical test ** Mann-Whitney U statistical

[a] children born in a non-EU country median 9 (min.1-max.15;IQR5) for externalizing behaviours

[b] 36.6% children born in a non-EU country did not received the routine health assessment for 4 year olds

For peer review only

Supplementary table 1b. Factors associated with the odds of developing emotional and behavioural difficulties at ages 4/5. Logistic regression model with immigrant status variable: 1st generation immigrant children.

	Adjusted Odds-Ratio	95% Confidence Interval	*p* value
**Variables**			
**Gender of the child**			
Boy	1.114	0.716–1.734	0.632
Girl	reference		
**Immigrant status**			
Child is 1st generation immigrant	2.247	1.062–4.756	0.034
Child is not 1st generation immigrant	reference		
**Household monthly income**			
<500 €	3.512	1.135–10.861	0.029
>500–750 €	1.968	0.705–492	0.196
>750–1000 €	1.947	0.682–5.562	0.213
>1000–1500 €	1.870	0.664–5.271	0.236
>1500–2000 €	1.531	0.469–5.001	0.480
>2000 €	reference		
**Parents Educational level**			
Lower education	2.995	1.303–6.884	0.010
9 years schooling	3.237	1.482–7.068	0.003
between 9 and 12 years	2.226	1.124–4.410	0.022
University degree	reference		

Supplementary table 1c. Psychomotor development at age 5

**Psychomotor development** n=107	Immigrant	Non-immigrant	Total	*P* value
Monitoring not required	37 (66.1)	40 (78.4)	77 (72.0)	0.155*
Monitoring required	19 (33.9)	11 (21.6)	30 (28.0)	

Significance level 5%. *Pearson- Chi square statistical test

For peer review only

Supplementary table 1b. Factors associated with the odds of developing emotional and behavioral problems immigrant status variable: 1st generation immigrant children

	aOR	95% CI	p value
Variables			
**Gender of the child**			
Boy	1.114	0.716— 1.734	0.632
Girl	reference		
**Immigrant status**			
Child is 1st generation immigrant	2.247	1.062—4.756	0.034
Child is not 1st generation immigrant	reference		
Household monthly income			
<500 €	3.512	1.135—10.861	0.029
>500-750 €	1.968	0.705—492	0.196
>750-1000€	1.947	0.682—5.562	0.213
>1000-1500€	1.870	0.664—5.271	0.236
>1500-2000€	1.531	0.469—5.001	0.480
>2000€	reference		
**Parents Educational level**			
Lower education	2.995	1.303—6.884	0.010
9 years schooling	3.237	1.482—7.068	0.003
between 9 and 12 years	2.226	1.124—4.410	0.022
University degree	reference		

For peer review only

Supplementary table 2. Fruit and vegetable intake and Body Mass Index (BMI) of children in the CRIAS cohort at ages 4/5

**Variables**	**Immigrant**	**Native**	**Total**	**p Value**
	n (%)	n (%)	n (%)	
	217 (51.7)	203 (48.3)	420 (100)	
**Dietary intake [a]**				
**Servings of fruit per day n=412**				<0.001*
Less than 2 servings/day	76 (36.0)	41 (20.4)	117 (27.9)	
2 or more servings/day	135 (64.0)	60 (79.6)	295 (70.2)	
**Servings of vegetables per day n=413**				<0.001*
Less than 3 servings/day	203 (95.8)	195 (97.0)	398 (94.8)	
3 or more servings/day	9 (4.2)	6 (3.0)	15 (3.6)	
**BMI n=314 [b]**				
**Overweight**				0.216*
Yes	35 (22.2)	44 (28.2)	79 (25.2)	
No	112 (71.8)	123 (77.8)	235 (74.8)	
**Obesity**				0.824*
Yes	9 (5.1)	8 (5.7)	17 (5.4)	
No	149 (94.9)	148 (94.3)	297 (94.6)	
**Underweight**				0.044*
Yes	18 (11.4)	8 (5.1)	26 (8.3)	
No	148 (88.6)	140	288 (91.7)	

Significance level 5%. *Pearson- Chi square statistical test

[a] intake according to recommend servings/day by the Portuguese Health Directorate
[b] underweight, overweight and obesity were classified using the World Health Organization Child Growth Standards charts

For peer review only

Supplementary table 3. Socioeconomic status and COVID-19 economic impact on families in the CRIAS-cohort study

	Non-Immigrants %	Immigrants %	Crude Odds-ratio (95% CI)	Adjusted Odds-ratio (95% CI)
**Education**				
Professional and higher education	30.9	17.1	1 [reference]	1 [reference]
Secondary education	35.5	38.8	2.0 (1.1-3.7)	1.18 (0.59-2.36)
Less than secondary education	33.6	44.0	2.4 (1.3-4.4)	0.89 (0.42-1.89)
**Employment**				
Employed	82.2	64.4	1 [reference]	1 [reference]
Unemployed and others	17.8	35.6	2.55 (1.48-4.41)	1.92 (1.03-3.55)
**Occupation**				
High-skilled occupations	81.6	57.8	1 [reference]	1 [reference]
Low-skilled occupations	18.4	42.2	3.24 (1.90-5.52)	2.49 (1.35-4.60)
**Family income before the pandemic**				
≥ 750 Euros	73.6	47.7	1 [reference]	1 [reference]
< 750 Euros	26.4	52.3	3.03 (1.84-5.09)	2.41 (1.36-4.28)
**Unemployed because of COVID-19**				
No	90.1	73.3	1 [reference]	1 [reference]
Yes	9.9	26.7	3.32 (1.72-6.40)	3.54 (1.72-7.30)
**On temporary or partial on lay-off because of COVID-19**				
No	82	51	1 [reference]	1 [reference]
Yes	49	69	2.26 (1.36-3.76)	2.09 (1.14-3.83)
**Household income change after the pandemic**				

For peer review only

Increased or remained the same	49.3	27.8	1 [reference]	1 [reference]
Decreased	50.7	72.2	2.53 (1.54-4.15)	3.21 (1.80-5.75)
**Falling behind with bills**				
No	76.3	59.7	1 [reference]	1 [reference]
Yes	23.7	40.3	2.18 (1.31-3.62)	1.95 (1.09-3.50)
**Financial difficulties in buying food**				
No	74.3	68.5	1 [reference]	1 [reference]
Yes	25.7	31.5	1.78 (1.08-2.94)	1.29 (0.72-2.30)
**Financial difficulties in buying hygiene products**				
No	77	57.5	1 [reference]	1 [reference]
Yes	23	42.5	2.47 (1.49-4.12)	1.95 (1.10-3.48)
**Financial difficulties to pay phone and internet**				
No	80.1	49.6	1 [reference]	1 [reference]
Yes	19.9	50.4	4.10 (2.42-6.92)	3.02 (1.65-5.53)
Kids go to school for a meal				
No	97.0	90.1	1 [reference]	1 [reference]
Yes	3.0	9.9	3.61 (1.14-11.37)	2.02 (0.57-7.19)

For peer review only

STROBE Statement—Checklist of items that should be included in reports of ***cohort studies***

	Item No	Recommendation	Page No
**Title and abstract**	1	(*a*) Indicate the study's design with a commonly used term in the title or the abstract	1
		(*b*) Provide in the abstract an informative and balanced summary of what was done and what was found	2
**Introduction**			
Background/rationale	2	Explain the scientific background and rationale for the investigation being reported	4
Objectives	3	State specific objectives, including any prespecified hypotheses	5
**Methods**			
Study design	4	Present key elements of study design early in the paper	6
Setting	5	Describe the setting, locations, and relevant dates, including periods of recruitment, exposure, follow-up, and data collection	6
Participants	6	(*a*) Give the eligibility criteria, and the sources and methods of selection of participants. Describe methods of follow-up	
		(*b*) For matched studies, give matching criteria and number of exposed and unexposed	7
Variables	7	Clearly define all outcomes, exposures, predictors, potential confounders, and effect modifiers. Give diagnostic criteria, if applicable	9-11
Data sources/ measurement	8*	For each variable of interest, give sources of data and details of methods of assessment (measurement). Describe comparability of assessment methods if there is more than one group	10
Bias	9	Describe any efforts to address potential sources of bias	7
Study size	10	Explain how the study size was arrived at	6
Quantitative variables	11	Explain how quantitative variables were handled in the analyses. If applicable, describe which groupings were chosen and why	9-11
Statistical methods	12	(*a*) Describe all statistical methods, including those used to control for confounding	
		(*b*) Describe any methods used to examine subgroups and interactions	7
		(*c*) Explain how missing data were addressed	
		(*d*) If applicable, explain how loss to follow-up was addressed	
		(*e*) Describe any sensitivity analyses	
**Results**			
Participants	13*	(a) Report numbers of individuals at each stage of study—eg numbers potentially eligible, examined for eligibility, confirmed eligible, included in the study, completing follow-up, and analysed	7
		(b) Give reasons for non-participation at each stage	
		(c) Consider use of a flow diagram	
Descriptive data	14*	(a) Give characteristics of study participants (eg demographic, clinical, social) and information on exposures and potential confounders	13
		(b) Indicate number of participants with missing data for each variable of interest	
		(c) Summarise follow-up time (eg, average and total amount)	
Outcome data	15*	Report numbers of outcome events or summary measures over time	13-18

For peer review only - http://bmjopen.bmj.com/site/about/guidelines.xhtml

Main results	16	(*a*) Give unadjusted estimates and, if applicable, confounder-adjusted estimates and their precision (eg, 95% confidence interval). Make clear which confounders were adjusted for and why they were included<br>(*b*) Report category boundaries when continuous variables were categorized<br>(*c*) If relevant, consider translating estimates of relative risk into absolute risk for a meaningful time period	13-18
Other analyses	17	Report other analyses done—eg analyses of subgroups and interactions, and sensitivity analyses	N/A
**Discussion**			
Key results	18	Summarise key results with reference to study objectives	N/A
Limitations	19	Discuss limitations of the study, taking into account sources of potential bias or imprecision. Discuss both direction and magnitude of any potential bias	19
Interpretation	20	Give a cautious overall interpretation of results considering objectives, limitations, multiplicity of analyses, results from similar studies, and other relevant evidence	N/A
Generalisability	21	Discuss the generalisability (external validity) of the study results	N/A
**Other information**			
Funding	22	Give the source of funding and the role of the funders for the present study and, if applicable, for the original study on which the present article is based	23

*Give information separately for exposed and unexposed groups.

Note: An Explanation and Elaboration article discusses each checklist item and gives methodological background and published examples of transparent reporting. The STROBE checklist is best used in conjunction with this article (freely available on the Web sites of PLoS Medicine at http://www.plosmedicine.org/, Annals of Internal Medicine at http://www.annals.org/, and Epidemiology at http://www.epidem.com/). Information on the STROBE Initiative is available at http://www.strobe-statement.org.

For peer review only

For peer review only - http://bmjopen.bmj.com/site/about/guidelines.xhtml
